# Incidence and consequences of damage to insecticide-treated mosquito nets in Kenya

**DOI:** 10.1186/s12936-021-03978-7

**Published:** 2021-12-20

**Authors:** Thomas Smith, Adrian Denz, Maurice Ombok, Nabie Bayoh, Hannah Koenker, Nakul Chitnis, Olivier Briet, Joshua Yukich, John E. Gimnig

**Affiliations:** 1 Swiss Tropical and Public Health Institute, 4051 Basel, Switzerland; 2 University of Basel, 4001 Basel, Switzerland; 3 Kenya Medical Research Institute (KEMRI), Kisumu, Kenya; 4 Tropical Health LLP, Baltimore, MD USA; 5 Tulane University School of Public Health and Tropical Medicine, New Orleans, LA USA; 6 Division of Parasitic Diseases and Malaria, Centers for Disease Control (CDC) and Prevention, Atlanta, GA USA

**Keywords:** Long-lasting insecticidal nets, Malaria

## Abstract

**Background:**

Efforts to improve the impact of long-lasting insecticidal nets (LLINs) should be informed by understanding of the causes of decay in effect. Holes in LLINs have been estimated to account for 7–11% of loss in effect on vectorial capacity for *Plasmodium falciparum* malaria in an analysis of repeated cross-sectional surveys of LLINs in Kenya. This does not account for the effect of holes as a cause of net attrition or non-use, which cannot be measured using only cross-sectional data. There is a need for estimates of how much these indirect effects of physical damage on use and attrition contribute to decay in effectiveness of LLINs.

**Methods:**

Use, physical integrity, and survival were assessed in a cohort of 4514 LLINs followed for up to 4 years in Kenya. Flow diagrams were used to illustrate how the status of nets, in terms of categories of use, physical integrity, and attrition, changed between surveys carried out at 6-month intervals. A compartment model defined in terms of ordinary differential equations (ODEs) was used to estimate the transition rates between the categories. Effects of physical damage to LLINs on use and attrition were quantified by simulating counterfactuals in which there was no damage.

**Results:**

Allowing for the direct effect of holes, the effect on use, and the effect on attrition, 18% of the impact on vectorial capacity was estimated to be lost because of damage. The estimated median lifetime of the LLINs was 2.9 years, but this was extended to 5.7 years in the counterfactual without physical damage. Nets that were in use were more likely to be in a damaged state than unused nets but use made little direct difference to LLIN lifetimes. Damage was reported as the reason for attrition for almost half of attrited nets, but the model estimated that almost all attrited nets had suffered some damage before attrition.

**Conclusions:**

Full quantification of the effects of damage will require measurement of the supply of new nets and of household stocks of unused nets, and also of their impacts on both net use and retention. The timing of mass distribution campaigns is less important than ensuring sufficient supply. In the Kenyan setting, nets acquired damage rapidly once use began and the damage led to rapid attrition. Increasing the robustness of nets could substantially increase their lifetime and impact but the impact of LLIN programmes on malaria transmission is ultimately limited by levels of use. Longitudinal analyses of net integrity data from different settings are needed to determine the importance of physical damage to nets as a driver of attrition and non-use, and the importance of frequent use as a cause of physical damage in different contexts.

**Supplementary Information:**

The online version contains supplementary material available at 10.1186/s12936-021-03978-7.

## Background

Insecticide-treated (bed) nets (ITNs) provide protection against malaria morbidity and mortality [1] and mass distribution of long-lasting insecticidal nets (LLINs) is the main preventive intervention accounting for the substantial reduction in global malaria burden seen in the last decade [2]. Based on World Health Organization (WHO) recommendations that LLINs should retain insecticidal activity for at least 3 years, national malaria control programmes distribute LLINs in 3-year cycles.

The WHO recommends that LLIN programmes implement monitoring activities both to assess the insecticidal activity and physical integrity of nets, and attrition under normal use conditions [3]. Cross-sectional assessment of surviving nets is the usual way of evaluating decay in insecticide, accrual of damage, and patterns of non-use (for instance [4–8]). These can provide a plethora of different outcomes, and it is not obvious what to prioritize. Rational planning of LLIN programmes would ideally use measurements of the contributions on a common scale of each of these different factors to decay in effectiveness. Mathematical models of the entomological effects of nets, parameterized using experimental hut data [7, 9], can help to achieve this by translating measurements of coverage, of decay in insecticide, and of physical integrity of nets, into effects on vectorial capacity. This composite variable has the important property of being independent of variations in malaria endemicity [10].

Partitioning of effects on vectorial capacity between different factors provides a means of identifying which improvements in LLINs will have the most impact. A recent analysis by Briet and colleagues of data from seven countries, where the US President’s Malaria Initiative (PMI) supported monitoring between 2009 and 2014, and of auxiliary data from experimental hut studies in Benin [11], estimated the impact of both physical and chemical decay on vectorial capacity [7]. Separate estimates of the contributions of different factors to loss of impact implied that variation in use generally has larger direct impacts than does attrition or physical damage, while effects of chemical decay are relatively small (assuming the levels of insecticide resistance in the field to be within the range tested in the experimental huts).

However, cross-sectional assessments alone do not reveal the full importance of each of these factors. A complete assessment of them should include both direct and indirect effects, which include the secondary effects of physical damage on attrition and non-use [12, 13]. Similarly, use of nets is essential for them to be effective, but also exposes them to greater risk of damage, so there are both direct and indirect effects of use. These indirect effects can only be estimated by linking repeated observations of the same nets. Since net use and physical damage to nets interact dynamically, a mathematical model incorporating these effects is needed to quantify the full impacts of both use and physical damage both on net survival and on vectorial capacity.

Previous mathematical models of the dynamics of LLIN populations [14–16] are stock-and-flow models of the distribution system, implicitly using simple representations of the life-histories of individual nets without considering which factors impact LLIN survival. When parameterized with data from national net cohorts which vary in the details of the monitoring programmes, and the types of LLINs included [17–19] they can be are used for imputing estimates of coverage, for quantifying net shortfalls, and for identifying replenishment needs.

This paper introduces models of the demography of nets incorporating the dynamic impacts of use and damage. These models are parameterized using the same net cohort data from Kenya as analysed by Briet and colleagues [7], which include survey rounds at 6-month intervals over 4 years, where both net use and physical integrity of nets were assessed. The life histories of these nets are analysed using a system of ordinary differential equations (ODEs) to model changes in status of nets during these intervals. The analysis provides estimates of the effects of physical damage to the LLINs on the probability that a net will be used, on the rate at which nets are discarded, and hence on the full lifetime impact on vectorial capacity. Comparisons with counterfactuals also provide estimates of how much use of nets contributes to decay in physical integrity and effectiveness. These quantities are estimated separately for the different net products in the Kenyan LLIN cohort.

## Methods

### Study site and cohort methodology

A total of 4514 nets were distributed to households in Gem sub-County, Siaya County, Kenya, in 2009. Each net was marked with a unique identifier. Nets of different types (different products of various brands) were distributed in the same area. Further details are provided elsewhere [7]. The studies in Kenya were selected for these analyses because assessments of physical integrity of the nets were carried out recurrently on some of the same nets both when they were reported as in use and when they were not in use. Data on the physical integrity of unused nets are essential for fitting the models.

Attrition, physical integrity and insecticidal activity of nets were assessed at 6-month intervals following the net distribution, with a series of eight surveys ending in 2014. At each visit, a questionnaire was administered to determine the presence/absence of the net and whether it was in use. Nets that were reported to be in use were further categorized according to whether they were in use the previous night. When nets were not present in the house, the questionnaire sought to determine the reason the net was absent. Net washing, and other factors potentially related to net durability (house type, number of residents, etc.) were also surveyed.

Physical integrity of nets refers to holes and tears in those nets still existing in households [20]. For the first five rounds a subset of randomly selected nets at each round was removed (target sample size 30 of each type) and analysed by counting holes and quantifying active ingredient in the laboratory. For surveys 6–8, a target of 50 nets of each type per round were analysed in the laboratory and holes were counted in the field in other nets. In each case, the measurements of holed area were converted to values of the WHO Pesticide Evaluation Scheme proportionate hole index (pHI) [21]. Nets were classified according to whether they were damaged or not based on a threshold value of pHI = 20, where this threshold value was determined empirically (see “Results” section). To avoid repeating the phrase ‘intact and lightly damaged,’ those nets with pHI < 20 are referred to in the remainder of the paper as ‘undamaged’.

### Classification of nets

Two different classifications of nets were used:

1. Classification by use
  At each survey with data, the nets were categorized as:
  i. New: this category applied only to the nets at the time of delivery.
  ii. Unused: where the respondents indicated that the nets were not used.
  iii. In use last night: Following standard practice in estimation of LLIN coverage, this category was used as the basis for estimating effective coverage.
  iv. In use, but not the previous night: For analyses of temporal dynamics, this category and the previous were aggregated (since no data were available on night-to-night changes in use which may by much more dynamic than changes in whether a net is reported to be in use at all).
  v. Attrited: Attrition is complete loss of nets, and can comprise both LLINs that are no longer usable or available (discarded, destroyed, repurposed) and those potentially still in use elsewhere (given away for others to use, sold, or stolen) [20]. For the standard, reference analyses (‘A1’), nets for which there was no information were included under attrition, while nets that were recorded as ‘elsewhere’ were not coded as attrited. Sensitivity analyses were carried out using variants of the WHO definition, namely (i) ‘A2’ coded only destroyed or repurposed nets under attrition; (ii) ‘A3’ also coded nets that were elsewhere as attrition; and (iii) ‘A4’ coded both nets that were elsewhere and those that with missing information as attrited. Details of these definitions are given in Additional file 1: Table S2.
  vi. If a net could not be assigned to one or other of these four categories it was omitted from the analysis.
2. Classification by use and physical integrity
  The classification according to use and physical integrity recognized six states:
  i. New: These nets (defined as above) are assumed to have been undamaged.
  ii. Undamaged unused: undamaged nets (pHI < 20), unused.
  iii. Undamaged used: undamaged nets (pHI < 20), in use.
  iv. Damaged used: damaged nets (pHI > 20), in use.
  v. Damaged unused: damaged nets (pHI > 20), unused.
  vi. Attrited: as defined in the classification by use.

Data on physical integrity were available for a much smaller subset of the survey records than was the data on use alone.

**Fig. 1:**
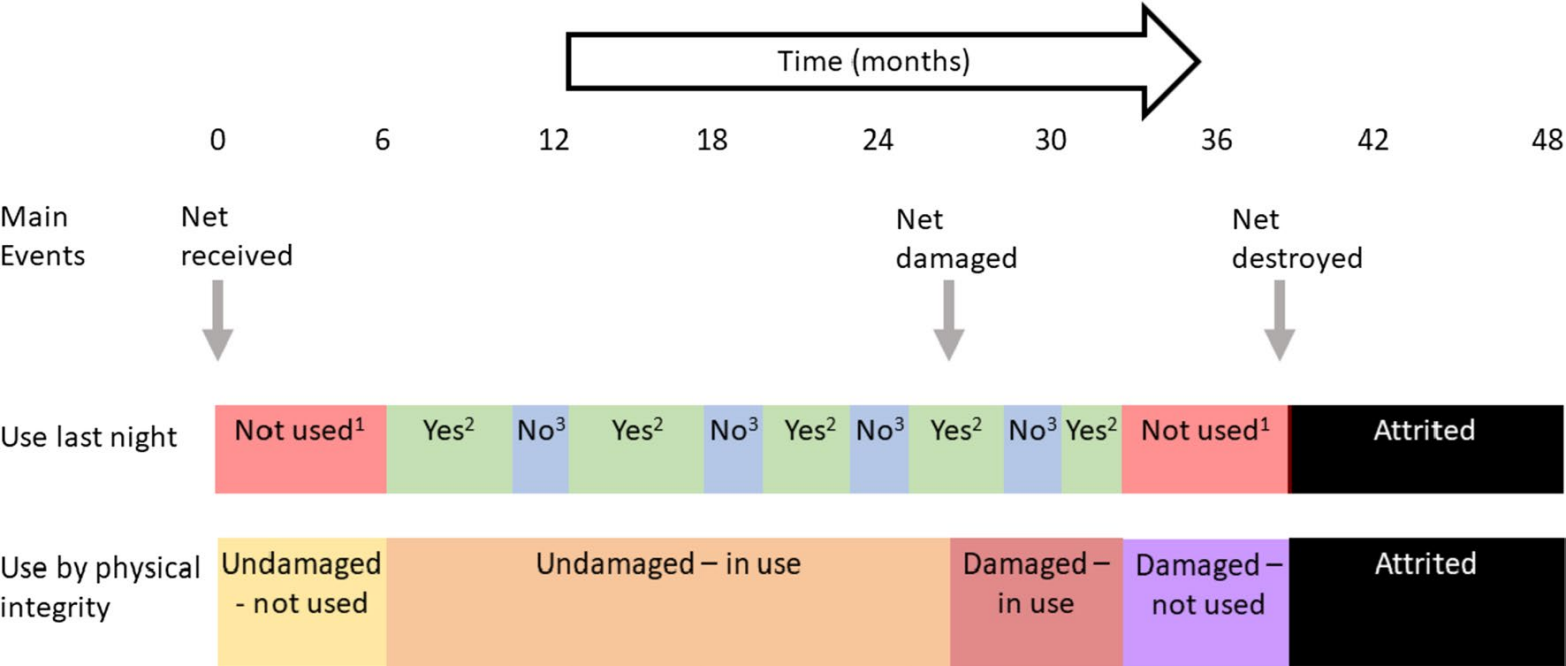
Hypothetical example life history of a single LLIN. Unused^1^: the net was recorded as not used; Yes^2^: the net was recorded as used ‘last night’; No^3^: the net was recorded as in use, but not on the previous night (see text for more explanation)

These classifications made it possible to assemble life-histories for individual nets for up to 48 months of the form shown in the hypothetical example of Fig. 1. The figure illustrates the life history of an example net, and how its status, in terms of both physical integrity and use, changes over time. The life history is conceptualized in terms of the events determining whether it is present, or damaged, which are treated as irreversible and occur in continuous time. Use status may also change in continuous time, with changes in use possible at any time that the net is present. Use-last-night can change from night to night but in Fig. 1 is imagined as changing every few months (no data were available on the timescale of changes in use-last-night). While the horizontal bars and events indicated by vertical arrows in Fig. 1 represent the life history in continuous time, this is observed only cross-sectionally at the 6-monthly survey time-points.

### Weighting of observations to allow for non-random loss of information

Changes in status were evaluated by cross-classifying the linked data from successive surveys. Average life histories of the whole net cohort were estimated by treating each 6-month inter-survey period as independent, and hence modelling the transitions (defined in terms of the physical integrity categories introduced in Fig. 1) as a first order Markov process. Only intervals of 6 months and with known initial and end status were included.

Many more intervals could be classified by use (Table 1) than by physical integrity. The different kinds of transitions were disproportionately represented in the classification by physical integrity because new or attrited nets could all be assigned to categories of physical integrity, while in the other categories, data on damage were available only for the subset of nets that had been examined. To account for this in the analyses that considered the extent of damage, the observations for each transition type (as defined using the classification that included physical integrity) were weighted depending on the categories of use (aggregating over categories of ‘use last night’). The weights were assigned so that the analysis that considered physical integrity employed the same relative frequencies of different transition-types defined by categories of use as were found in the overall dataset.

**Table 1:** Numbers of nets with data for each survey and category

	Survey month							
	6	12	18	24	30	36	42	48
Total nets present	4069	3845	3571	3460	2782	2610	1891	1515
In use last night	2614	2530	2643	2587	1954	1573	1109	841
In use but not last night	349	472	301	306	200	333	119	270
Not in use	1106	843	627	567	628	704	663	404
Elsewhere^a^	278	345	358	429	725	812	828	838
Recorded as attrition^a^	36	29	47	100	159	233	390	609
Total assessed for physical integrity	205	206	209	206	752	896	784	601
Damaged and in use	36	50	98	91	312	381	354	340
Damaged and not in use	7	11	11	20	57	136	179	130
Undamaged and in use	122	103	75	82	300	287	157	131
Undamaged and not in use	40	42	25	13	83	92	94	57
Physical integrity assessed at previous survey^b^	205^c^	0	0	0	0	178	207	171

^a^Using the definitions applied in analysis A1

^b^Number of nets assessed for physical integrity that were also assessed 6 months previously

^c^New nets are assumed to be undamaged and hence counted as assessed at baseline

### Differential equation model

**Fig. 2:**
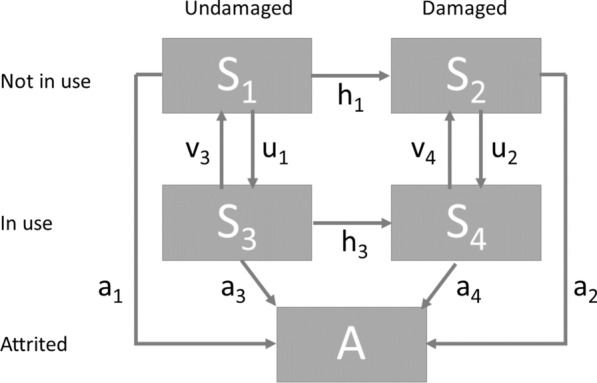
Compartments and parameters of the ODE model

Five net states were considered; ${\mathrm{S}}_{1}$: undamaged nets not in use; ${\mathrm{S}}_{2}$: damaged nets not in use; ${\mathrm{S}}_{3}$: undamaged nets in use; ${\mathrm{S}}_{4}$: damaged nets in use; $\mathrm{A}$: attrited nets. New nets were assigned to state ${\mathrm{S}}_{1}$. The analysis did not consider whether the nets were recorded as in use the previous night (since this can change in both directions from night-to-night and data are only available for observations at 6-month intervals). The proportions of the net cohort in each category, at time t, are related via a system of differential equations (Fig. 2):

$$\frac{{\mathrm{dS}}_{1}}{\mathrm{dt}}={\mathrm{v}}_{3}{\mathrm{S}}_{3}-\left({\mathrm{u}}_{1}+{\mathrm{h}}_{1}+{\mathrm{a}}_{1}\right){\mathrm{S}}_{1}$$

$$\frac{{\mathrm{dS}}_{2}}{\mathrm{dt}}={\mathrm{h}}_{1}{\mathrm{S}}_{1}+{\mathrm{v}}_{4}{\mathrm{S}}_{4}-\left({\mathrm{u}}_{2}+{\mathrm{a}}_{2}\right){\mathrm{S}}_{2}$$

$$\frac{{\mathrm{dS}}_{3}}{\mathrm{dt}}={\mathrm{u}}_{1}{\mathrm{S}}_{1}-\left({\mathrm{v}}_{3}+{\mathrm{h}}_{3}+{\mathrm{a}}_{3}\right){\mathrm{S}}_{3}$$

$$\frac{{\mathrm{dS}}_{4}}{\mathrm{dt}}={\mathrm{h}}_{3}{\mathrm{S}}_{3}+{\mathrm{u}}_{2}{\mathrm{S}}_{2}-\left({\mathrm{v}}_{4}+{\mathrm{a}}_{4}\right){\mathrm{S}}_{4}$$

$$\frac{\mathrm{dA}}{\mathrm{dt}}{=\mathrm{a}}_{1}{\mathrm{S}}_{1}{+\mathrm{ a}}_{2}{\mathrm{S}}_{2}{+\mathrm{ a}}_{3}{\mathrm{S}}_{3}{+\mathrm{ a}}_{4}{\mathrm{S}}_{4}$$

where the parameters are: ${{\mathrm{a}}_{1}\dots \mathrm{a}}_{4}$ attrition rates of states 1…4, respectively; ${{\mathrm{h}}_{1},\mathrm{h}}_{3}$: rates at which nets in states 1 and 3 respectively become damaged; ${{\mathrm{u}}_{1},\mathrm{u}}_{2}$: rates at which nets in states 1 and 2 are taken into use; ${{\mathrm{v}}_{3},\mathrm{v}}_{4}$: rates at which nets in states 3 and 4 fall out of use. Damaged nets are expected to be more likely to be discarded, i.e. ${{\mathrm{a}}_{4}>\mathrm{a}}_{3}$ and ${{\mathrm{a}}_{2}>\mathrm{a}}_{1}$; and more likely to fall out of use, i.e. ${{\mathrm{v}}_{4}>\mathrm{v}}_{3}$; conversely, nets in use are anticipated to acquire holes more rapidly, i.e. ${{\mathrm{h}}_{3}>\mathrm{h}}_{1}$. The proportions of nets in the system are constrained by the requirement that the total of the proportions for the different states must add up to one, and the initial conditions (when the nets have just been delivered and there are no holes) are ${\mathrm{S}}_{1}{+\mathrm{ S}}_{3}=1$, with proportions in the other states initialized as zero. This allows for uncertainty in ${\mathrm{S}}_{3}(0)$, the proportion of nets taken into use immediately on deployment. During an interval, nets can transition in both integrity and use but the probability that both changes occur at exactly the same time is zero.

The rate of repair of nets was constrained to be zero, since from the perspective of this study, partially repaired nets do not change category and continue to be included among those that are damaged and the small number of nets that was recorded as transitioning from the damaged to undamaged categories were treated as data errors. For the primary analysis, these nets were recoded as damaged throughout the interval. Sensitivity analyses in which these nets were coded as initially undamaged are reported in detail in Additional file 1.

### Fitting to observed transition probabilities

The ODE system gave an expression for the proportions of nets in each of the categories ${\mathrm{S}}_{1}\dots {\mathrm{ S}}_{4}$ and the absorbing state, $\mathrm{A}$, as a function, $\mathrm{F}\left(\mathrm{t},\mathrm{p},\mathrm{S}\right)$ where t is the time (in years) since the previous survey, p is the parameter vector ${{\{\mathrm{h}}_{1},\mathrm{h}}_{3} {{\mathrm{ u}}_{1},\mathrm{u}}_{2}{{\mathrm{ v}}_{3},\mathrm{v}}_{4},{\mathrm{ a}}_{1},{\mathrm{ a}}_{2},{\mathrm{ a}}_{3},{\mathrm{a}}_{4}$}, and S = {${\mathrm{S}}_{1}\left(0\right)\dots .{\mathrm{ S}}_{4}$(0), A(0)} is the vector of state probabilities at the start of the interval ($\mathrm{t}=0$).

Bayesian estimates of p were obtained by jointly fitting to the physical integrity categories from the first cross-sectional survey, and to the state transitions aggregated over all the other 6-month inter-survey intervals. Fitting was carried out in the R-Stan R-package [22] using a gradient-based Markov chain Monte Carlo (MCMC) algorithm [23], with imprecise log-normal priors for each of the rates.

At the time of distribution the expected proportions of LLINs in each state are given by the initial status vector $\mathrm{S}\left(0\right)=\left\{{ {\mathrm{S}}_{1}\left(0\right)=1-\mathrm{U}}_{\mathrm{N}}, {\mathrm{S}}_{2}\left(0\right)=0,{ {\mathrm{S}}_{3}\left(0\right)=\mathrm{U}}_{\mathrm{N}}, {\mathrm{S}}_{4}\left(0\right)=0,\mathrm{ A}\left(0\right)=0\right\}$, where ${\mathrm{U}}_{\mathrm{N}}$ is the proportion of new nets that go immediately into use. (Initial analyses attempted to estimate ${\mathrm{U}}_{\mathrm{N}}$, but the posterior density for this parameter concentrated on the lower bound of the $\mathrm{Uniform}(\mathrm{0,1})$ prior, implying that a model with a fixed value of ${\mathrm{U}}_{\mathrm{N}}=0$ was more appropriate. The subsequent analyses reported in detail adopted a fixed value of ${\mathrm{U}}_{\mathrm{N}}=0$). The observed numbers of nets in each state at the first survey were assumed to be multinomially distributed about $\mathrm{F}\left(0.5,\mathrm{p},\mathrm{S}(0)\right)$.

For surveys at time-points beyond 6 months, the model was used to predict separately the multinomial proportions of LLINs in each state at the end of the interval, conditioning on each of the possible initial states. Thus, the expected probabilities are given by $\mathrm{F}\left(\mathrm{t},\mathrm{p},\mathrm{S}\right)$ where the initial state is $\mathrm{S}(\mathrm{t}-0.5)$.

### Estimation of derived quantities

The forward Euler method was used to provide solutions of the ODEs for each of a sample of 1000 draws from the joint posterior distribution of the model parameters with the initial status vector $\mathrm{S}\left(0\right)=\left\{{\mathrm{S}}_{1}\left(0\right)=1, {\mathrm{S}}_{2}\left(0\right)=0,{\mathrm{S}}_{3}\left(0\right)=0, {\mathrm{S}}_{4}\left(0\right)=0,\mathrm{ A}\left(0\right)=0\right\}$, corresponding to ${\mathrm{U}}_{\mathrm{N}}=0$, the case where newly delivered nets all go into the unused category. These solutions provided point and interval estimates of the ODE model parameters (Table 2) and derived quantities (Table 3). The latter were calculated as functions of the estimated parameters of the ODE model and point estimates of the probabilities that in-use damaged and undamaged nets were used the night before the survey, calculated directly from the survey data. In addition to the simulations carried out using the fitted estimates of the parameters of the ODE model, these calculations required two counterfactual scenarios corresponding to ideal situations to which a programme might aspire. In the counterfactual simulation denoted ‘no damage’, the rates at which nets acquire holes, ${\mathrm{h}}_{1}$ and ${\mathrm{h}}_{3}$ (and therefore ${\mathrm{S}}_{2}$ and ${\mathrm{S}}_{4}$) were set to zero. In the counterfactual denoted ‘full use’, all the nets were in use until attrited, so new nets were assigned to ${\mathrm{S}}_{3}$ and ${\mathrm{v}}_{3}$ and ${\mathrm{v}}_{4}$ were both set to zero. In each case, the other parameters of the ODE remained at their fitted values. The no-damage counterfactual thus had a longer median lifetime, ${\mathrm{L}}_{50}^{(\mathrm{H}=0)}$ (where H indicates holes) because of the lower attrition rates of undamaged nets. Comparison of forward simulations of these counterfactuals with the forward simulation based on the estimated rates of use and damage provided estimates for the impact of damage on use and attrition.

**Table 2:** Parameter estimates from the model fitted to the full database (analysis A1)

Symbol	Explanation	Units	Estimate (95% credible interval)
${\mathbf{h}}_{1}$	Acquisition of holes in unused nets	Year^a^	1.18 (0.98, 1.42)
${\mathbf{h}}_{3}$	Acquisition of holes in nets in use	Year^a^	0.87 (0.76, 0.98)
${\mathbf{u}}_{1}$	Putting undamaged nets to use	Year^a^	1.67 (1.40, 2.01)
${\mathbf{u}}_{2}$	Putting damaged nets to use	Year^a^	1.30 (1.13, 1.47)
${\mathbf{v}}_{3}$	Taking undamaged nets out of use	Year^a^	2.77 (2.45, 3.22)
${\mathbf{v}}_{4}$	Taking damaged nets out of use	Year^a^	0.44 (0.39, 0.51)
${\mathbf{a}}_{1}$	Attrition of undamaged, unused nets	Year^a^	0.04 (0.01, 0.09)
${\mathbf{a}}_{2}$	Attrition of damaged, unused nets	Year^a^	0.37 (0.29, 0.47)
${\mathbf{a}}_{3}$	Attrition of undamaged, used nets	Year^a^	0.26 (0.20, 0.33)
${\mathbf{a}}_{4}$	Attrition of damaged, used nets	Year^a^	0.26 (0.23, 0.30)
${\mathbf{P}}_{\mathbf{o}}$	Proportion of nets at follow-up for which physical integrity was evaluated	Probability	0.60 (0.59, 0.61)
${\mathbf{U}}_{\mathbf{N}}$	Probability a new net is taken into use immediately on receipt	Probability	0.00 (fixed)
${\mathbf{U}}_{\mathbf{H}}$	Probability a damaged net was used the night before survey (if nets is ‘in use’)^a^	Probability	0.94
${\mathbf{U}}_{\mathbf{I}}$	Probability an undamaged net was used the night before survey (if net is ‘in use’)^a^	Probability	0.88

Note that a rate, $\mathrm{h}$ gives rise to a probability $1-\mathrm{ exp}(-\mathrm{ht}$) that the event occurs in $\mathrm{t}$ years, ignoring competing events

^a^These proportions obtained directly from the survey data

**Table 3:** Estimates of derived quantities (analysis A1, with 95% credible intervals)

Quantity			Estimate (95% credible interval)
Median life of LLIN (years)	${\mathrm{L}}_{50}$		2.86 (2.68, 3.08)
Proportion of lifetime of LLIN for which it is in use^a^	${\mathrm{P}}_{\mathrm{U}1}$	$\left({\int }_{\mathrm{t}=0}^{\infty }{\mathrm{S}}_{3}\left(\mathrm{t}\right)+ {\mathrm{S}}_{4}\left(\mathrm{t}\right)\mathrm{ dt}\right)/\left({\int }_{\mathrm{t}=0}^{\infty }1-\mathrm{A}\left(\mathrm{t}\right)\mathrm{ dt}\right)$	0.60 (0.58, 0.62)
Proportion of lifetime of LLIN for which it is damaged	${\mathrm{P}}_{\mathrm{H}}$	$\left({\int }_{\mathrm{t}=0}^{\infty }{\mathrm{S}}_{2}\left(\mathrm{t}\right)+ {\mathrm{S}}_{4}\left(\mathrm{t}\right)\mathrm{ dt}\right)/\left({\int }_{\mathrm{t}=0}^{\infty }1-\mathrm{A}\left(\mathrm{t}\right)\mathrm{ dt}\right)$	0.78 (0.75, 0.81)
Proportion of lifetime of LLIN for which it is in use^b^	${\mathrm{P}}_{\mathrm{U}2}$	$\left({\int }_{\mathrm{t}=0}^{\infty }{{\mathrm{U}}_{\mathrm{I}}\mathrm{S}}_{3}\left(\mathrm{t}\right)+ {\mathrm{U}}_{\mathrm{H}}{\mathrm{S}}_{4}\left(\mathrm{t}\right)\mathrm{ dt}\right)/\left({\int }_{\mathrm{t}=0}^{\infty }1-\mathrm{A}\left(\mathrm{t}\right)\mathrm{ dt}\right)$	0.56 (0.54, 0.58)
Reduction in net lifetime attributable to holes (years)	${\mathrm{L}}_{\mathrm{H}}$	${\mathrm{L}}_{50}^{(\mathrm{H}=0)}-{\mathrm{L}}_{50}$	2.88 (1.77, 4.34)
Proportion loss in net lifetime attributable to holes	${\mathrm{P}}_{\mathrm{H}}$	$1-{\int }_{\mathrm{t}=0}^{\infty }1-\mathrm{A}\left(\mathrm{t}\right)\mathrm{ dt}/{\int }_{\mathrm{t}=0}^{\infty }1-{\mathrm{A}}^{(\mathrm{H}=0)}\left(\mathrm{t}\right)\mathrm{ dt}$	0.49 (0.38, 0.58)
Proportion of attrition in damaged nets	${\mathrm{P}}_{\mathrm{AH}}$	${\int }_{\mathrm{t}=0}^{\infty } {\mathrm{a}}_{2}{\mathrm{S}}_{2}\left(\mathrm{t}\right)+ {\mathrm{a}}_{4}{\mathrm{S}}_{4}\left(\mathrm{t}\right)\mathrm{ dt}/{\int }_{\mathrm{t}=0}^{\infty }{\mathrm{a}}_{1}{\mathrm{S}}_{1}\left(\mathrm{t}\right)+ {\mathrm{a}}_{2}{\mathrm{S}}_{2}\left(\mathrm{t}\right)+{\mathrm{a}}_{3}{\mathrm{S}}_{3}\left(\mathrm{t}\right)+ {\mathrm{a}}_{4}{\mathrm{S}}_{4}\left(\mathrm{t}\right)\mathrm{ dt}$	0.91 (0.88, 0.93)
Proportion of reduced use^a^ attributable to holes	${\mathrm{P}}_{\mathrm{UH}1}$	$\frac{{\int }_{\mathrm{t}=0}^{\infty }{{\mathrm{S}}_{3}\left(\mathrm{t}\right)+ {\mathrm{S}}_{4}\left(\mathrm{t}\right)-\mathrm{S}}_{3}^{(\mathrm{H}=0)}\left(\mathrm{t}\right)- {\mathrm{S}}_{4}^{(\mathrm{H}=0)}\left(\mathrm{t}\right)\mathrm{ dt}}{1-{\int }_{\mathrm{t}=0}^{\infty }{\mathrm{S}}_{3}^{(\mathrm{H}=0)}\left(\mathrm{t}\right)+ {\mathrm{S}}_{4}^{(\mathrm{H}=0)}\left(\mathrm{t}\right)\mathrm{ dt}}$	0.13 (− 0.08, 0.30)
Proportion of reduced use^b^ attributable to holes	${\mathrm{P}}_{\mathrm{UH}2}$	$\frac{{\int }_{\mathrm{t}=0}^{\infty }{\mathrm{U}}_{\mathrm{I}}\left({\mathrm{S}}_{3}\left(\mathrm{t}\right)-{\mathrm{S}}_{3}^{(\mathrm{H}=0)}\left(\mathrm{t}\right)\right)+ {\mathrm{U}}_{\mathrm{H}}\left({\mathrm{S}}_{4}\left(\mathrm{t}\right)- {\mathrm{S}}_{4}^{(\mathrm{H}=0)}\left(\mathrm{t}\right)\right)\mathrm{ dt}}{1-{\int }_{\mathrm{t}=0}^{\infty }{{\mathrm{U}}_{\mathrm{I}}\mathrm{S}}_{3}^{(\mathrm{H}=0)}\left(\mathrm{t}\right)+ {{\mathrm{U}}_{\mathrm{H}}\mathrm{S}}_{4}^{(\mathrm{H}=0)}\left(\mathrm{t}\right)\mathrm{ dt}}$	0.09 (− 0.13, 0.27)
Total nights in use^b^	${\mathrm{L}}_{\mathrm{U}}$	$365 {\mathrm{L}}_{50}{\mathrm{P}}_{\mathrm{U}2}$	581 (529, 639)
Total nights use of a net resistant to damage	${\mathrm{L}}_{\mathrm{U}}^{(\mathrm{H}=0)}$	$365 {\mathrm{L}}_{50}^{(\mathrm{H}=0)}{\mathrm{P}}_{\mathrm{U}2}$	654 (530, 823)
Proportion of the theoretical maximal use, lost because of damage	${\mathrm{X}}_{\mathrm{H}}$	$1-\frac{{\mathrm{L}}_{\mathrm{U}}}{{\mathrm{L}}_{\mathrm{U}}^{(\mathrm{H}=0)}}$	0.10 (− 0.12, 0.32)
Proportion of impact on vectorial capacity lost because of damage^c,d^	${\mathrm{X}}_{\mathrm{V}}$	$1-\frac{\left(1-{\mathrm{P}}_{\mathrm{V}}\right){\mathrm{L}}_{\mathrm{U}}}{{\mathrm{L}}_{\mathrm{U}}^{(\mathrm{H}=0)}}$	0.18 (− 0.03, 0.38)

^a^As defined by ‘net in use’

^b^As defined by ‘net in use last night’

^c^Including the reduction in available nets due to attrition via its effect on ${\mathrm{L}}_{\mathrm{U}}$

^d^Where ${\mathrm{P}}_{\mathrm{V}}=0.08$ is the estimate of the proportion of impact on vectorial capacity lost because of the direct effect of holes in nets

To estimate the overall impact of damage on the ability of nets to reduce malaria transmission, a value of ${\mathrm{P}}_{\mathrm{V}}=0.08$ was used for the proportion of potential impact on vectorial capacity lost because of the direct effect of holes in nets. The previous analysis that linked the observed levels of damage in the Kenyan net cohort to experimental hut data on the entomological effects of holes in LLINs [7] gave values of ${\mathrm{P}}_{\mathrm{V}}=0.11$ and of ${\mathrm{P}}_{\mathrm{V}}=0.07$ for Kenya for susceptible and resistant mosquitoes, respectively. A value of ${\mathrm{P}}_{\mathrm{V}}=0.08$ was used as an approximation for this quantity to represent the high frequency of resistance in contemporary Kenyan anopheline populations, which implies that the value should be closer to that based on data for the resistant laboratory colony.

### Estimation of net-type specific quantities

The overall Kenyan net cohort includes seven different product types with the numbers of intervals analysed for each net type varying between 726 and 998 (Table 4). After fitting the model to the full dataset, the ODE model was fitted separately to the data for each specific net type using the standard definition of attrition (A1).

**Table 4:** Numbers of intervals analysed by net type

Dawaplus^®^ 2.0	DuraNet©	Interceptor^®^	NetProtect^®^	Olyset™	PermaNet^®^ 2.0	PermaNet^®^ 3.0
791	998	902	856	788	726	861

### Analysis of reports of reasons for attrition

Reporting by the householder of whether an attrited net was discarded because it was damaged is logically distinct from both (i) whether it was already damaged before the end of life (because even a damaged net might be discarded for some other reason), and (ii) whether pre-existing damage was a cause of the attrition (since the owner might say for instance that they decided to repurpose the net, or that they did not like the design, without indicating that this was because of damage).

At each survey at time $\mathrm{t}$, questionnaire responses on whether the net was attrited because of damage were available for a total of ${\mathrm{n}}_{\mathrm{x}}\left(\mathrm{t}\right)$ nets attrited in the preceding interval, with ${\mathrm{r}}_{\mathrm{x}}\left(\mathrm{t}\right)$ reported to have been attrited because of damage, providing an estimate ${\widehat{{\mathrm{P}}_{\mathrm{x}}}\left(\mathrm{t}\right)=\mathrm{r}}_{\mathrm{x}}\left(\mathrm{t}\right)/{\mathrm{n}}_{\mathrm{x}}\left(\mathrm{t}\right)$ of the proportion, ${\mathrm{P}}_{\mathrm{x}}\left(\mathrm{t}\right)$, of attrition that is considered by the householders as being due to damage. It is proposed that the overall proportion of all attrition where the net was reported to be damaged (irrespective of whether it was reported discarded because of damage), can be related to pre-existing damage via the relationship:

$$\begin{array}{c}{\mathrm{P}}_{\mathrm{x}}\left(\mathrm{t}\right)=\frac{{\mathrm{a}}_{\mathrm{c}}+ {\mathrm{a}}_{\mathrm{i}}{\mathrm{P}}_{\mathrm{d}}\left(\mathrm{t}\right){\mathrm{P}}_{\mathrm{ix}}}{\mathrm{a}\left(\mathrm{t}\right)}\, {\text{f}}{\text{o}}{\text{r}}\, 1 \le i \le 4,\end{array}$$

where ${\mathrm{a}}_{\mathrm{c}}$ is the rate of attrition due to ‘catastrophic’ damage (meaning the net would have been attrited anyway, irrespective of whether it was previously damaged); ${\mathrm{a}}_{\mathrm{i}}$ is the rate of attrition via processes that are incremental to previous damage; ${\mathrm{P}}_{\mathrm{ix}}$ is the proportion of this incremental attrition that is reported by the householder as being due to damage, ${\mathrm{P}}_{\mathrm{d}}\left(\mathrm{t}\right)={\mathrm{S}}_{2}\left(\mathrm{t}\right)+{\mathrm{S}}_{4}\left(\mathrm{t}\right)$ is the proportion of the extant net cohort that is damaged at time t (available from the forward simulation of the ODE analysis A1), and $\mathrm{a}\left(\mathrm{t}\right)={\mathrm{ a}}_{1}{\mathrm{S}}_{1}\left(\mathrm{t}\right)+{\mathrm{ a}}_{2}{\mathrm{S}}_{2}\left(\mathrm{t}\right)+{\mathrm{ a}}_{3}{\mathrm{S}}_{3}\left(\mathrm{t}\right)+{\mathrm{ a}}_{4}{\mathrm{S}}_{4}\left(\mathrm{t}\right)$ is the overall attrition rate, also available from the ODE.

At time 0, ${\mathrm{S}}_{1}\left(0\right)=1$; $\mathrm{a}\left(0\right)={\mathrm{a}}_{1}$; ${\mathrm{P}}_{\mathrm{d}}\left(0\right)=0$, and hence ${\mathrm{P}}_{\mathrm{x}}\left(0\right)=\frac{{\mathrm{a}}_{\mathrm{c}}}{{\mathrm{a}}_{1}}$; and ${\mathrm{a}}_{1}={\mathrm{a}}_{\mathrm{u}}+ {\mathrm{a}}_{\mathrm{c}}$, where ${\mathrm{a}}_{1}$ is the attrition rate of new, unused, undamaged nets (see Table 5); and ${\mathrm{a}}_{\mathrm{u}}$ is the initial rate of attrition that is unrelated to either pre-existing or catastrophic damage. This makes it possible to specify constraints on ${\mathrm{a}}_{\mathrm{c}}:0<{\mathrm{a}}_{\mathrm{c}}<{\mathrm{a}}_{1}$, and since ${\mathrm{a}}_{\mathrm{i}}$ is constrained to be less than the total attrition rate in damaged nets (less the attrition that would have happened anyway) at all values of t, ${\mathrm{a}}_{\mathrm{i}}: 0<{\mathrm{a}}_{\mathrm{i}}<\mathrm{min}\left(\frac{\mathrm{a}\left(\mathrm{t}\right)-{\mathrm{a}}_{\mathrm{c}}}{{\mathrm{P}}_{\mathrm{d}}\left(\mathrm{t}\right)}\right)$.

**Table 5:** Parameter estimates from analysis of reported reasons for attrition

Parameter	Prior distribution	Description	Estimate (95% credible interval)
${\mathrm{a}}_{\mathrm{c}}$	$\mathrm{Uniform}(0,{\mathrm{a}}_{1})$	Rate of attrition by catastrophic damage	0.002 (0.0001, 0.010)
${\mathrm{a}}_{\mathrm{i}}$	$\mathrm{Uniform}(0,\mathrm{min}\left(\frac{\mathrm{a}\left(\mathrm{t}\right)-{\mathrm{a}}_{\mathrm{c}}}{{\mathrm{P}}_{\mathrm{d}}\left(\mathrm{t}\right)}\right))$	Rate of attrition by incremental damage	0.20 (0.14, 0.30)
${\mathrm{P}}_{\mathrm{ix}}$	$\mathrm{Uniform}(\mathrm{0,1})$	Proportion of incremental attrition reported as due to damage	0.69 (0.47, 0.97)

A Bayesian model (fitted in rjags) was then used to estimate ${\mathrm{a}}_{\mathrm{c}}, {\mathrm{a}}_{\mathrm{i}}$ and ${\mathrm{P}}_{\mathrm{ix}}$, conditional on the point estimates of $\mathrm{a}\left(\mathrm{t}\right), {\mathrm{a}}_{1}$ and ${\mathrm{P}}_{\mathrm{d}}\left(\mathrm{t}\right)$ from the ODE, by assuming the number of nets reported as attrited due to damage at each survey to be binomially distributed:

$${\mathrm{r}}_{\mathrm{x}}\left(\mathrm{t}\right)\sim \mathrm{Binomial}\left({{\mathrm{n}}_{\mathrm{x}}\left(\mathrm{t}\right),\mathrm{P}}_{\mathrm{x}}\left(\mathrm{t}\right)\right)$$

where the quantities used in the formula for ${\mathrm{P}}_{\mathrm{x}}\left(\mathrm{t}\right)$ are computed as means over the 6-month interval preceding the survey. This model provided the parameter estimates given in Table 5. At each survey time point, the nets attrited during the preceding interval can then be assigned to the categories listed in Table 6, with the proportions assigned to each category calculated using simple probability calculus.

**Table 6:** Categorization of attrited nets by physical integrity at time point of attrition

Pre-existing damage	Attrition reported as due to damage	Proportion of attrition
Yes	Yes	${\mathrm{P}}_{\mathrm{x}}\left(\mathrm{t}\right)-{\mathrm{a}}_{\mathrm{c}}\left(1-{\mathrm{P}}_{\mathrm{d}}\left(\mathrm{t}\right)\right)/\mathrm{a}\left(\mathrm{t}\right)$
No	Yes	${\mathrm{a}}_{\mathrm{c}}\left(1-{\mathrm{P}}_{\mathrm{d}}\left(\mathrm{t}\right)\right)/\mathrm{a}\left(\mathrm{t}\right)$
Yes	No	${\mathrm{P}}_{\mathrm{d}}\left(\mathrm{t}\right)+\frac{\left({\mathrm{a}}_{\mathrm{c}}+{\mathrm{a}}_{\mathrm{i}}{\mathrm{P}}_{\mathrm{d}}\left(\mathrm{t}\right)\right)}{\mathrm{a}\left(\mathrm{t}\right)}-{\mathrm{P}}_{\mathrm{x}}\left(\mathrm{t}\right)$
No	No	$\left(1-\frac{\left({\mathrm{a}}_{\mathrm{c}}+{\mathrm{a}}_{\mathrm{i}}{\mathrm{P}}_{\mathrm{d}}\left(\mathrm{t}\right)\right)}{\mathrm{a}\left(\mathrm{t}\right)}\right)\left(1-{\mathrm{P}}_{\mathrm{d}}\left(\mathrm{t}\right)\right)$

## Results

The parameter estimates from the ODE model are given in Table 2, and the estimates of the key derived quantities, including the time that nets spend in different states are given in Table 3. The relationships of these quantities to the data, and the interpretation of the numbers of nets in different categories and of the transition probabilities, is complicated because the data arise from competing processes that lead to the nets in each category being non-random subsets of the total. While use of nets is expected to result in damage, this is not evident as a correlation of damage and use in the data. Among the records with physical integrity data that were included in the analyses, 309/543 (57%) of nets in use were in a damaged state (as defined using the pHI threshold of 20), compared with 118/211 (56%) of the nets that were not in use. Among nets with physical integrity data at successive surveys, 71/482 (15%) of new or unused nets became damaged during the interval (Table 7), a near-identical percentage to that among nets that were in use at the start of the interval (107/727). Perhaps surprisingly, the model estimate of ${\mathrm{h}}_{3}$, the rate of transition from intact to holed in nets in use, was lower than that of ${\mathrm{h}}_{1}$, the rate of transition from intact to holed in unused nets (Table 2).

**Table 7:** Numbers of intervals analysed by physical integrity

Initial status	Status at end of interval							
	Attrition	Damaged-in use	Damaged-not in use	Undamaged-in use	Undamaged-not in use	NA-not in use	NA-in use	Total
New	36	35	7	116	40	1059	2812	4105
Damaged-in use	582	156	37	*34*	*7*	58	209	1083
Damaged-not in use	154	19	37	*1*	*7*	64	29	311
Undamaged-in use	537	85	22	66	17	63	191	981
Undamaged-not in use	180	14	15	17	22	46	37	331
Total	1489	309	118	234	93	1290	3278	6811

Undamaged = pHI < 20; Attrition according to definition A1; NA = Integrity status not available. The values in italic correspond to LLINs recorded as transitioning from damaged to undamaged, that were treated as remaining damaged for the purpose of fitting the ODE models

The number of nets examined at each survey declined over time (Table 1) as previously described [7]. The nets included in the surveys of physical integrity were only a small proportion of the cohort, with the main variation by survey round relating to a change in protocol at 30 months (Table 1). At the first four surveys, holes were only counted in removed nets, so longitudinal data on net integrity was available only for the first interval (since the nets could all be assumed to be initially undamaged) and from the last three intervals.

**Fig. 3:**
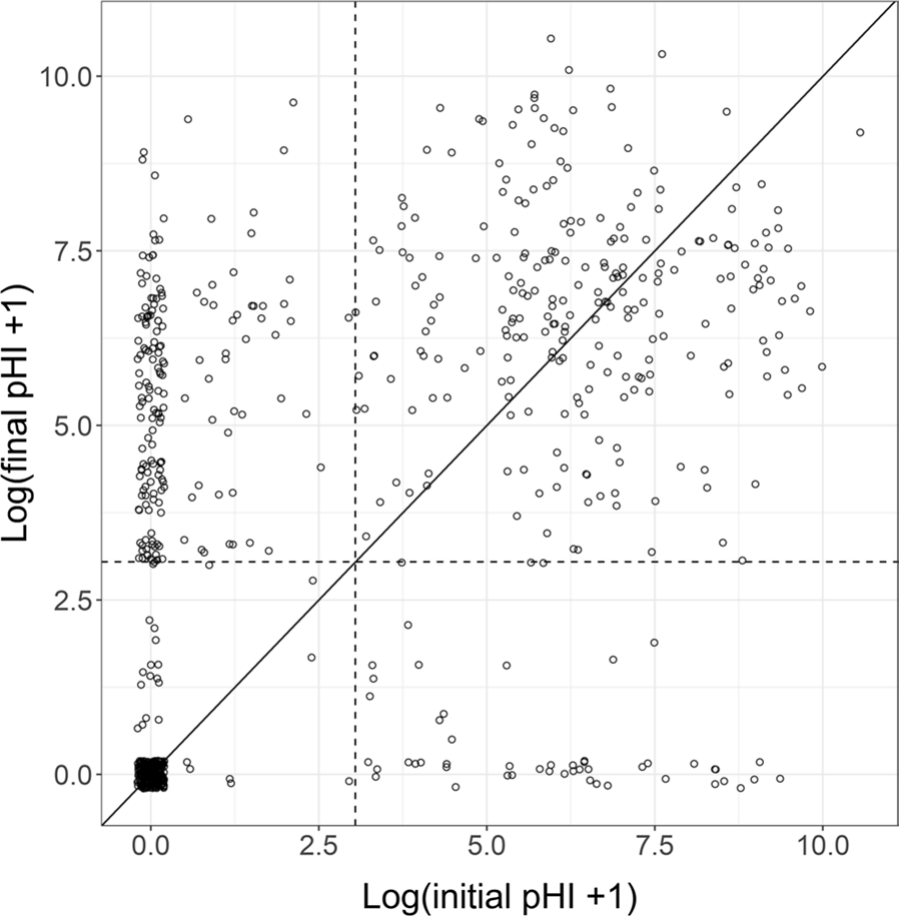
Proportionate Hole Index (pHI): scatter of final vs initial values at 6-month intervals. The diagonal line corresponds to no change in physical integrity of the net. Points are jiggered to minimize overprinting. The black square in the bottom left corresponds to nets that were reported undamaged at both the start and end of the interval. The dashed lines correspond to pHI of 20

Few nets transition from damaged to undamaged. Among intervals where physical integrity was assessed both at the start and end, in 429/761 (56%) of instances, the pHI at the end of the interval was greater than at the start. In 164 (22%) of instances, the reported pHI decreased (Fig. 3). 145 (36%) intervals where the net initially had no holes ended with pHI > 0, while in 258 instances nets recorded as pHI = 0, were also recorded 6-months later as pHI = 0. The pHI threshold of 20 (the dashed lines in Fig. 3) represents a very small area of holes and was chosen to minimize counts of nets that appear to have been repaired (those italicised in Table 7). Most of these apparent improvements probably result from measurement error and do not represent meaningful repair.

**Fig. 4:**
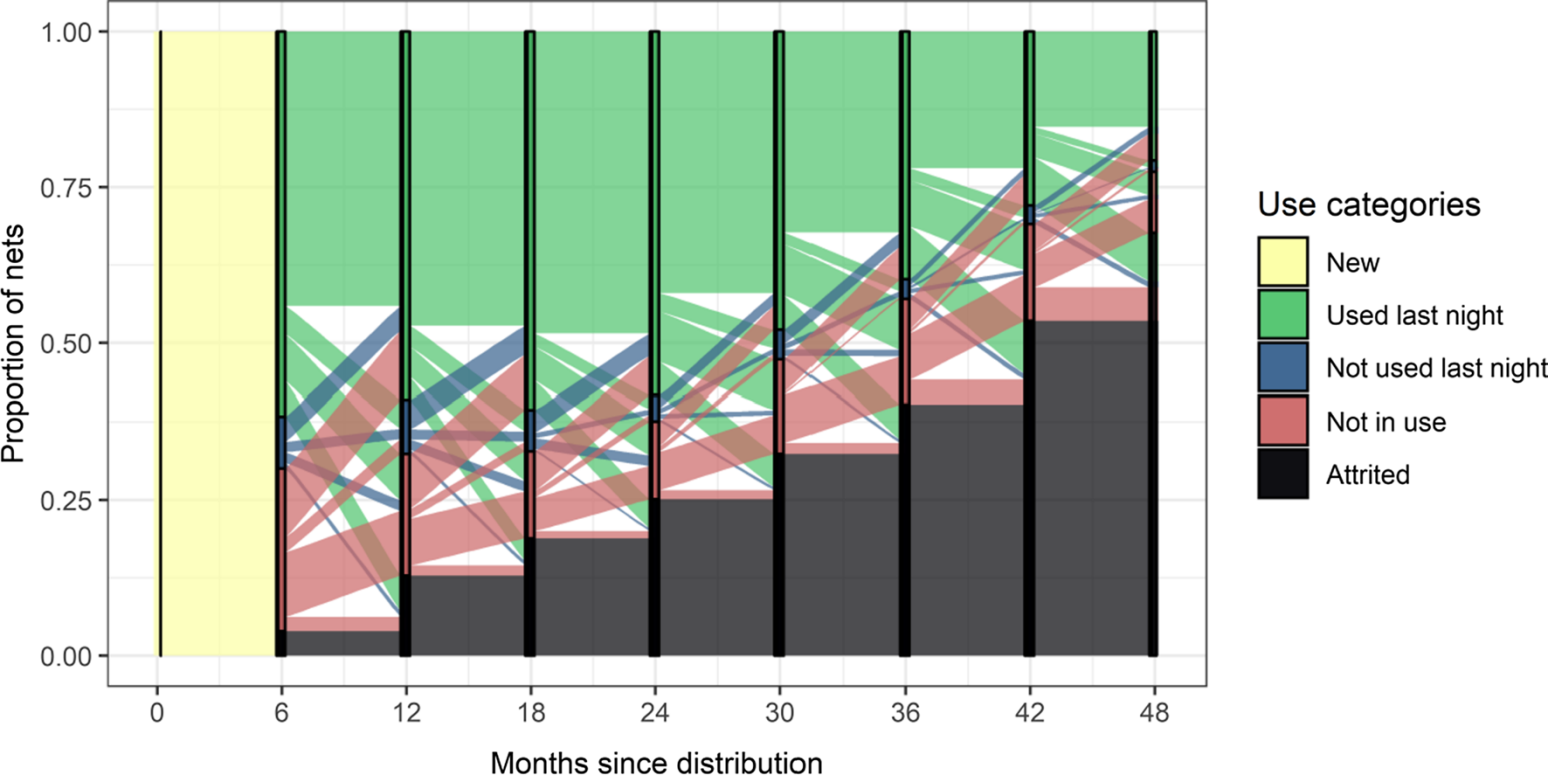
Observed flows between categories of use. The vertical bars indicate the times of the initial net distribution and of the surveys and the status at survey; the coloured bands connecting surveys indicate transition flows where the colors indicate the status of the nets at the previous survey; the height of the bands corresponds to the proportion of nets undergoing the transitions indicated among all survey intervals with both initial and final status defined. The transition probabilities between categories were calculated separately for each inter-survey interval. Attrited was defined as for analysis A1

**Fig. 5:**
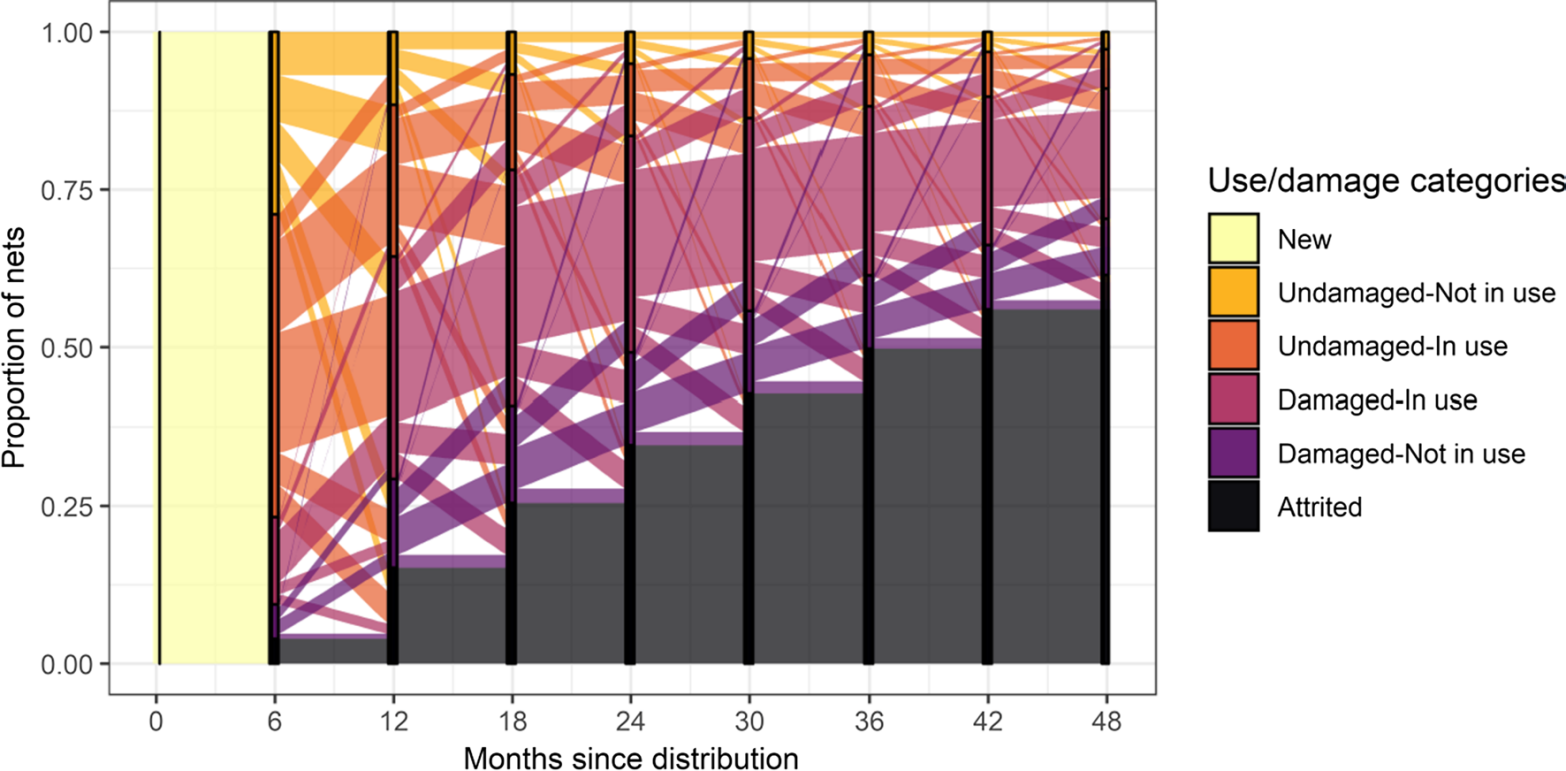
Observed flows between categories defined by physical integrity and use based on Markov approximation. Explanation of vertical bands and colored bands as for Fig. 4. Data for transitions for which the physical integrity of the net was assessed at the start of the interval weighted by frequencies of transitions by categories of use. Common transition probabilities between categories were used for all inter-survey intervals (analysis A1), with the data on physical integrity weighted so that the transition probabilities between categories of use correspond to those in the overall dataset

The life histories of nets were initially summarized as flows between categories of use (Fig. 4) and attrition, with the latter an absorbing class into which nets flow at the end of their lives (although not all nets had reached this stage by the end of the study). Attrition of nets occurs gradually, rather than after a fixed lifetime. The flows between ‘use last night’ and ‘use, but not last night’, are substantial in both directions, which is consistent with the understanding that many nets are used seasonally or occasionally. The available data do not directly indicate how frequently nets are taken into use immediately upon reception but the number of nets in the not-in-use category is relatively high at 6 months, then decreases, but increases again at the end of the follow-up. This is partly explained by the analysis of flows by net integrity (Fig. 5). As the nets age, they generally flow from the ‘undamaged unused’ state, to ‘undamaged, in use’ then ‘Damaged in use’ and finally ‘Damaged unused’, with this latter category relatively infrequent. Nets that were in use were more likely to be damaged than unused nets (compare the ratios of different categories in Fig. 5) with this ratio increasing with the age of the net.

Figures 4 and 5 show broadly similar patterns of use over time, although Fig. 5 was constructed by assigning the same transition probabilities for each 6 month interval (instead of separate values for each age-category of net), calculated by aggregating data for each type of transition across all survey intervals (Table 7). This implies that it is reasonable to approximate the transitions by a first-order Markov process, in which the age of a net does not directly influence use, damage or attrition, but rather that these occur at age-independent rates (like the decay of radioactive particles), with the accumulation of nets in the damaged class resulting in an increase over time in the attrition rate. However, Fig. 4 suggests that the proportion of the cohort that is attrited increases more-or-less linearly with time, while in Fig. 5 there is a suggestion that the proportion attrited is a concave function of the age of the net, which implies that there is a small deviation from the Markov approximation.

The ODE model makes use of this Markov assumption, applying the same parameter estimates (Table 2) to the whole of the life histories. In an initial fit, ${\mathrm{U}}_{\mathrm{N}}$, the proportion of new nets that go immediately into use was also included in the estimation, however the posterior density for this parameter concentrated on the lower bound of the $\mathrm{Uniform}(\mathrm{0,1})$ prior, implying that a model with a fixed value of ${\mathrm{U}}_{\mathrm{N}}=0$ was more appropriate and that the nets were rarely put to use immediately upon receipt. The other parameters were estimated with reasonably narrow intervals (Table 2), with nets estimated to be moving in and out of use much more rapidly than they are destroyed or lost (the rates of attrition are all lower than the rates of moving in or out of use). This was irrespective of whether the nets were used last night or not, which is not considered in this model.

**Fig. 6:**
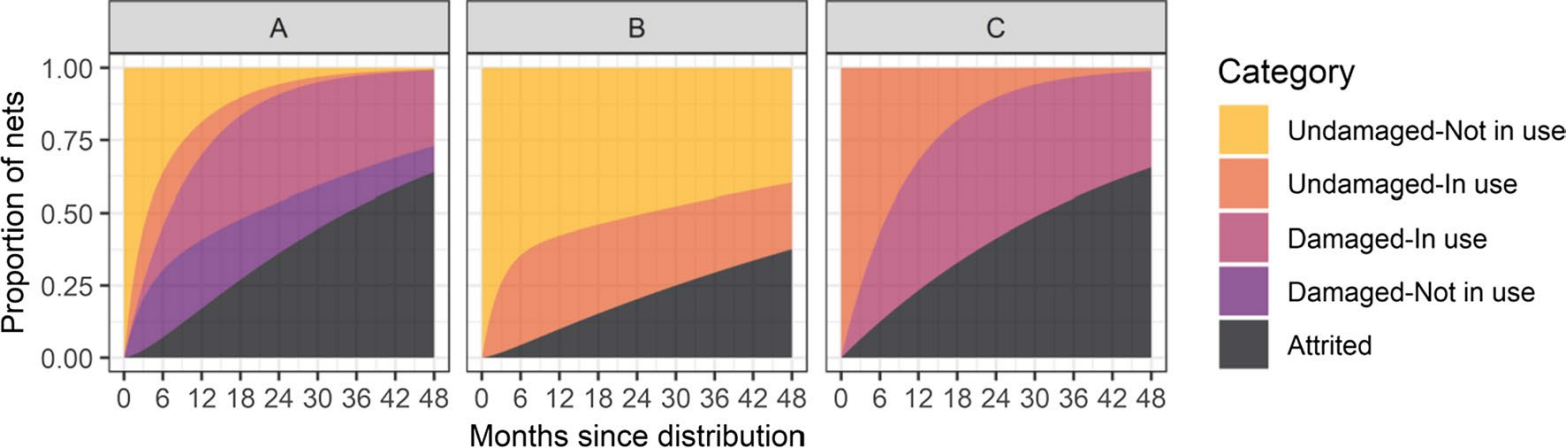
Projections from the reference model (A1) and counterfactuals. **A** Fitted parameter values; **B** no-damage counterfactual; **C** ‘full use’ counterfactual

The MCMC algorithm gives quite narrow interval estimates for all the parameters (Table 2) but there are strong correlations between some pairs of estimates (Additional file 1: Fig. S1). In principle, longitudinal data enable simultaneous estimation of transition rates from use back to non-use, as well as from non-use to use. However, while the steady-state transition rates between categories of use, ${\mathrm{u}}_{1}/{\mathrm{ v}}_{3}$ and ${\mathrm{u}}_{2}/{\mathrm{ v}}_{4}$ can be estimated quite precisely: undamaged nets (${\mathrm{u}}_{1}/{\mathrm{ v}}_{3}=1.32$, 95% credible interval: 1.20, 1.44); damaged nets (${\mathrm{u}}_{2}/{\mathrm{ v}}_{4}=2.06$, 95% credible interval: 1.80, 2.36) the individual estimates of ${\mathrm{u}}_{1}$ and ${\mathrm{v}}_{3}$ are very highly correlated with each other, and ${\mathrm{u}}_{2}$ is strongly correlated with ${\mathrm{v}}_{4}$. The estimated flow of undamaged nets into use expressed either as the single parameter ${\mathrm{u}}_{1}$, or the ratio ${\mathrm{u}}_{1}/{\mathrm{ v}}_{3}$ is thus lower than that of damaged ones (${\mathrm{u}}_{2}$ or ${\mathrm{u}}_{2}/{\mathrm{ v}}_{4}$), (Table 2) consistent with new (undamaged) nets spending considerable time stored before being taken into use. This partly explains the lack of positive correlation between damage and use, and means that simulations of hypothetical no-damage nets (Fig. 6B), predict that such nets would spend a lot of time in the ‘not used’ category, so that at use at 36 months is only about 25% (Fig. 6B) when damage is precluded compared with the 38% observed (Fig. 6A). However, this is based on the conservative assumption that hypothetical undamageable nets would mostly be stored away.

Forward simulation of the ODEs, using the fitted parameter values provides estimates of the survival of the nets (Table 3) and the proportion of nets in each category in continuous time (Fig. 6A). The estimated median lifetime of the LLINs was 2.86 years (95% credible interval: 2.68, 3.08). These estimates are broadly coherent with the observations plotted in Fig. 5, though at 6 months only about 22% of nets appear as damaged defined by categories of use in Fig. 5 compared with about 38% in Fig. 6A. Conversely, in the oldest nets the observed percentage damaged is slightly lower than the fitted values. This indicates that though the model broadly reproduces the patterns in the data, rates of accrual of damage show a tendency to increase as nets age, again implying that the Markov approximation is a simplification.

**Fig. 7:**
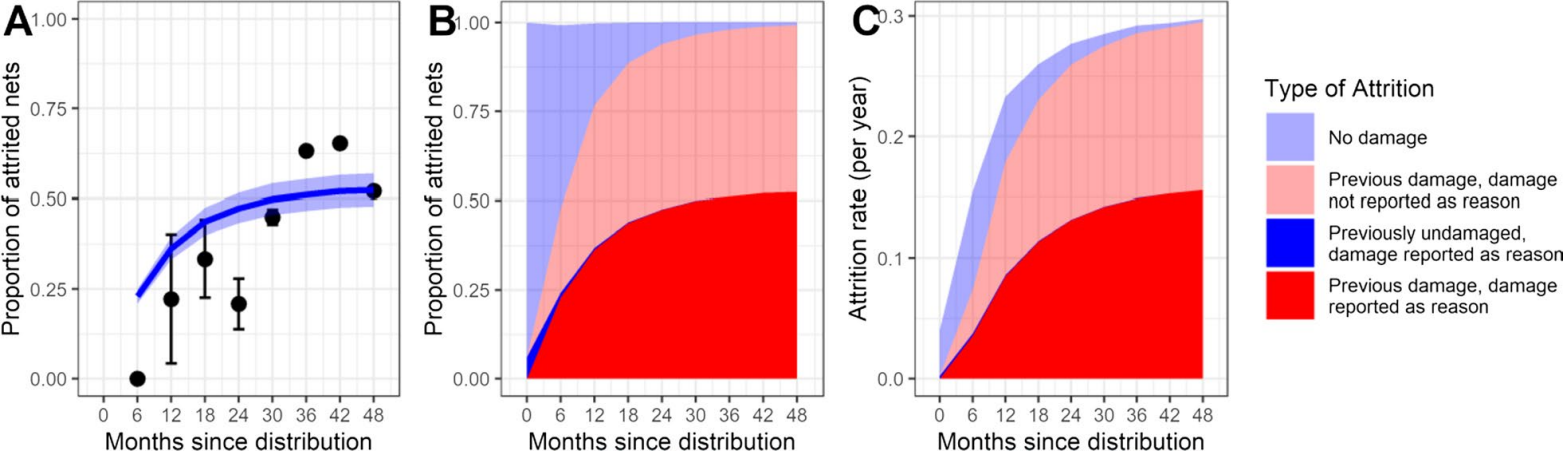
Estimates by net product type. **A** Parameters of the ODE model; **B** derived proportions; **C** estimates of durations from ODE model. The grey bars represent 95% credible intervals for the estimates derived from the overall dataset. ‘Lifetime in use’ refers to the proportion of the time from deployment to attrition for which the net is in use: ^1^as defined by ‘net in use’; ^2^as defined by ‘net in use last night’

Levels of use are estimated to have little overall effect on rates of attrition. This is because overall attrition of unused nets arises as an average of very low attrition of undamaged nets (${\mathrm{a}}_{1}$), and very high attrition (${\mathrm{a}}_{3}$) among unused, but damaged nets, which are likely to be ones that have been taken out of use because of the damage, in preparation for repurposing or disposal. Consequently, the counterfactual with full use of nets (Fig. 6C) has a similar rate of attrition to that in the data and in Fig. 6A. Related to this, the proportion of underuse that can be attributed to physical damage to the nets (Fig. 7, Table 3, and Additional file 1: Table S6) is low.

In the ODE model, the overall survival of a net is gamma-distributed, resulting from convolution of exponential distributions. This leads to a rather more gradual increase in the proportion attrited than is seen with the functional forms fitted to attrition over time in previous analyses of net durability [24], albeit one that matches reasonably well the pattern observed in the data (Fig. 4). The estimate of the median life of an LLIN is close to the widely assumed value of 3 years, but it is in use for a total number of 581 nights (95% credible interval 529–639 days, Table 3). In the counterfactual analysis without physical damage the lifetime is roughly doubled, to 5.7 years (${\mathrm{L}}_{\mathrm{H}}$ = 2.88 (1.77, 4.34), Table 3), but this is partly because the nets spend much of their lifetime in the unused state, so this translates into less than a doubling in the use that is obtained from each net. The estimate of the overall loss of use because of damage is consequently much less than the reduction in the lifetime, however the interval estimates for the estimated percentage loss in use linked to physical damage are very broad, ranging from − 12 to 32% (Table 3) reflecting sensitivity to the difference in use by category of net integrity. The estimate of this quantity is based on relatively little data, and might well change if nets were more robust, since this would very likely change the availability of nets and hence the rate at which they were taken into use.

The estimates of damage and loss of use (including the effect of reduction in available nets due to attrition) were converted into estimates of the effect on vectorial capacity by adjusting for the direct effect on vectorial capacity of holes in nets, computed for the same net cohort (Table 3). This gave an estimate that 18% (CI − 3–38%) of the impact on vectorial capacity is lost because of damage, when all three effects are considered: the direct effect, the effect on use, and the effect on attrition. The forward simulations of the models with different definitions of attrition (Additional file 1: Table S2) are presented in Additional file 1: Table S5 as are the detailed results of the comparisons of different net product types, and these illustrate the sensitivity of the parameter estimates and also the estimates of net survival parameters (Additional file 1: Table S6) to these definitions. The results of the sensitivity analyses are described in detail in Additional file 1. Additional file 1: Tables S5 and S6 give the detailed results of the comparisons of different net product types, and these also provide the sensitivity of the parameter estimates and the estimates of net survival parameters to the classification of use-status. There is considerable variation between net types, with approximately two-fold variation in the median lifetime between longest and shortest lifetime brands, but with most of the confidence intervals overlapping (Additional file 1: Table S6).

**Fig. 8:**
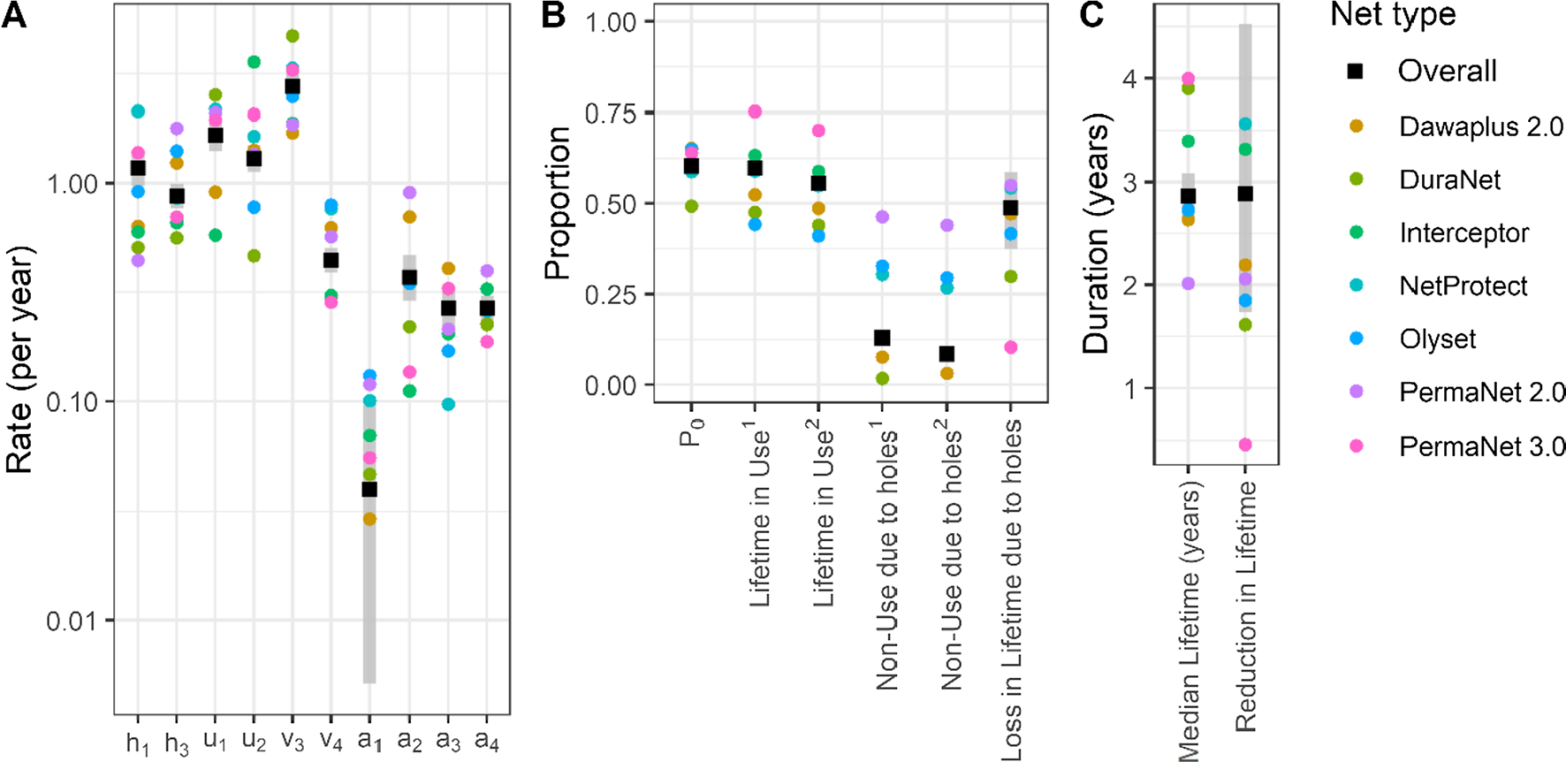
Attrition by reported reason over time. **A** Self-reporting of the proportion of nets attrited because of damage, by survey. Error bars are 95% binomial confidence intervals, the blue line is the fit from the regression model of reported damage as a function of pre-existing damage, as estimated from the ODE; Shading indicates 95% credible interval; **B** Estimated proportion of attrited nets in each category, values for time points between surveys are linear interpolations, categories are as elaborated in Table 6. **C** Results from panel **B** expressed as rates (obtained by multiplying the proportions by the overall attrition rate, $\mathrm{a}\left(\mathrm{t}\right)$)

Of those attrited nets with responses to the question about the reason for attrition, 44% (706/1603) were reported to have been attrited because of damage (Additional file 1: Table S1). This proportion increased strongly with the age of the nets, with 0/36 responses at 6 months reporting damage as the reason (Fig. 8A). The visual check suggests that the model that fitted this proportion as a function of the proportion of damaged nets gave a reasonable fit for later time points but could not fit to this low value because much of the attrition, even at 6 months, is estimated to be of nets that are previously damaged (Fig. 8B), albeit since the previous survey. An even steeper increase with net age in the proportion reported as damaged would be anticipated if this trend reflects only the increase in the proportion of damaged nets in the cohort.

The model estimated that the rate of catastrophic destruction of nets (i.e. destruction that is independent of pre-existing damage) is very low (Table 5). Among older nets, a very large proportion of the attrition is in nets with pre-existing damage (Fig. 8B) suggesting that although most attrition of damaged nets is described as due to damage by the householder (Table 5) even when the householders do not report attrition as being due to damage, the net is likely to have had pre-existing damage. Since the most of the attrited nets that were not reported as torn or damaged were reported to be burnt, it appears that younger nets are burnt relatively more frequently than the older ones, and that most of those that are burnt are already damaged (Additional file 1: Table S1).

## Discussion

Physical damage to mosquito nets is a widely recognized problem, and in the context of evidence that the global burden of malaria disease is no longer decreasing [25], there are concerns that many of the LLINs currently being deployed may have useful lifetimes much shorter than 3 years. Levels of pyrethroid resistance in malaria vectors are increasing [26], and there have been considerable efforts to develop LLINs treated with additional non-pyrethroid insecticides, or synergists. Reduction in effectiveness against malaria transmission results from both loss of insecticidal activity and physical damage to nets though there is considerable evidence that the physical durability of LLINs is the primary factor limiting their longevity [13, 18, 27–30]. LLIN owners may stop using, discard, or repurpose damaged nets, even if the nets still have some effectiveness against malaria transmission. If LLIN distribution rates are insufficient to make sure new LLINs are available in the household to replace disused nets, this could lead to temporary resurgences in malaria in between distribution rounds.

It is challenging to fully quantify the public health importance of damage to nets, because it has both direct and indirect consequences. Damage results in reductions in deterrence and killing of mosquitoes that have direct effects on malaria transmission that can be estimated using experimental hut data [7], but the health effects in any specific place also depend on the transmission intensity and the coverage of nets in the population. Cross-sectional surveys provide rich data on how many nets are in use and their physical state, but not on the (greater) likelihood of damaged nets being destroyed or on the relationships between damage and use. Longitudinal analyses of the histories of individual mosquito nets are needed to measure these secondary effects, and a comprehensive understanding needs to consider physical integrity data for representative samples of both nets in use, and those not in use. Few monitoring programmes can provide all these data. In the recently reported multi-country net cohorts [7], data on physical integrity were collected only for a small sample of the unused nets, so the present analyses are necessarily heavily dependent on data from the small proportion of the nets that were monitored. In interpreting these results, the possibility that the Kenyan net cohort may not be very typical should be borne in mind: it is likely that awareness of the study contributed to better-than-average care of nets (a Hawthorne effect). Survival of the nets in Kenya was also better than in the other cohorts included in that analysis. The full set of data needs to be collected for other net cohorts to be able to assess the generalizability of the results.

Flow diagrams (Figs. 4 and 5) describe how the status at the end of an interval depends on that at the start and illustrate the broad pattern of the life histories of individual nets, but indicate nothing about the causes of non-use or attrition. LLINs are generally taken into use during the first 6 months of ownership but rarely immediately on receipt [31]. Once damaged, they generally continue to be used for a period before being taken out of use, but after that they are soon discarded, so few nets from the study cohort are found in a damaged, but unused state (correspondingly, the model estimates of ${\mathrm{a}}_{2}>{\mathrm{a}}_{3}$ and ${\mathrm{a}}_{2}>{\mathrm{a}}_{4}$). The flow diagrams are agnostic about the exact times when transitions occur during the intervals and the same net can experience multiple events in the same interval, so if an initially undamaged net has been discarded by the time of the survey at the end of the interval, it might have been damaged at some point in between, but this possible event does not appear in the diagram. Similarly, a net recorded at both surveys as unused could have acquired holes during a transient period of use, or during storage, for example due to rodents.

The ODE model infers how much of the attrition and non-use is attributable to physical damage, extending the logic of other studies that have estimated the direct transmission impact of holes in nets, or for how much of the lifetime of a net it is damaged [7, 24]. The approximation that about 8% of the loss of impact on vectorial capacity (see “Methods”) is accounted for by physical damage in extant nets is based on a maximum lifetime of 3 years. The present analysis suggests that median net lifetime is similar to 3 years and this similarity appears to support net deployment at this interval, since this achieves a steady-state in coverage. However, this matching may be no coincidence not only because the deployment interval influences product specifications but also because distribution of new nets may act as a trigger for disposal of older, damaged ones. If there were no physical damage, nets would have almost twice this lifetime, with a median survival of about 5.7 years (Table 3). The analysis of the retrospectively reported reasons for attrition also suggests that roughly half of the attrited nets are disposed of because of damage (though almost all attrited nets have some measurable damage, even if this was not cited as the reason for disposal). More robust nets would likely be more expensive, but because they would need to be replaced less frequently this would not necessarily add to programme costs. This is coherent with recent advocacy for nets that are more durable even if untreated [32].

Some simplifications are unavoidable in the ODE modelling. These include the Markov assumption common to ODE modelling (equivalent to constant failure rates of given categories of nets), and the exclusion of rare states and transitions from the system illustrated in Fig. 2. Multiple state-transitions can occur in the same interval using rates defined in continuous time, but the inclusion of the full set of possible transitions would lead to a more complicated model with identifiability problems. Specifically, field studies frequently find partial repairs to nets but even when there is a substantial effort to repair nets, it is unusual for all the holes to be repaired [33–35]. Consistent with this, few nets appear in the data to move from the damaged to undamaged states, and the ODE model assumes that this does not happen.

Tools already exist for providing programmes with relevant information on the state of the existing net population, the level of insecticide resistance, the costs and durability of new nets, and the local malaria epidemiology. Net robustness and effects of physical damage on attrition should also be considered when deciding how frequently to replenish LLINs, and which brand of net should be provided. There is relatively good information on coverage gaps from high-resolution spatiotemporal coverage and use estimates derived using statistical models of the relationship between net coverage, use, ownership and other standard ITN indicators [2].

Coverage is also frequently modelled as a function of supply and rates of median net survival using population models of ITN distribution systems, parameterized using procurement, distribution, and cross-sectional survey data, albeit without accounting for how inflow of new nets influences survival of extant nets. Such models include the stock and flow compartment model of Flaxman and colleagues [15], and NetCALC [14] which tracks numbers of nets in the population by age category. Empirical models of net survival and predictions of public-health impacts [7, 24, 36] can be used to improve on allocation decisions based on the standard assumed net lifetime of 3 years. There have also been efforts to better characterize the physical integrity of LLINs using resistance to damage (RD) scores [37] to predict LLIN rates of attrition and degradation under field conditions. Though not yet adopted as a tool for LLIN procurement, RD scores aim is to optimize LLIN selection as well as provide manufacturers with a framework to develop and evaluate novel products. The current analysis suggests that such efforts to improve the physical durability of LLINs could have substantial impacts on LLIN longevity and use. However, rates of attrition, of use, and of both chemical and physical decay of LLINs in the PMI dataset vary widely and in unexplained ways both between net product types and countries [7, 31] and the dynamics of holes and their effects might well be equally variable. It is not clear how much the results of the present analysis are applicable to other settings or scenarios.

Physical damage to nets can be a reason why nets are unused, but many nets continue to be used despite being damaged, and in the Kenyan cohort, damaged nets are more frequently in use than undamaged ones. This is because damaged nets are soon discarded or repurposed, while undamaged nets spend more time in the unused state. The undamaged ones go more rapidly into use (${\mathrm{u}}_{1}>{\mathrm{u}}_{2}$), they return sooner into the unused stock (${\mathrm{v}}_{3}>{\mathrm{v}}_{4}$) and have relatively low attrition when not in use. Damaged ones are less likely to be taken down and back into unused stock and are much more likely to be attrited (${\mathrm{a}}_{2}>{\mathrm{a}}_{1}$). It is likely that this reflects the rapid acquisition of damage once nets go into use and prompt disposal once the owner decides a net is no longer serviceable. A consequence is that in the counterfactual of Fig. 6B, where nets do not acquire damage, there is lower average use per extant net than in the status quo simulation. If both the lower use of undamaged nets and the direct entomological effects of holes are taken into account, an individual net is estimated to achieve about 82% of the impact it would have if there was no physical damage. This 18% loss of impact is much less than the halving implied by the net survival estimates on their own. However, there are various reasons for supposing this to be a very conservative estimate. If people intending to sleep under nets have a sufficient supply of them, then making nets more robust would also be associated with lower rates at which they are taken into use. Distribution rates would presumably be reduced to avoid accumulation of unused nets. The counterfactual of Fig. 6B is thus only a first-order approximation of what would happen if nets were replaced with nets that last longer. It is very unlikely that hypothetical ‘undamageable’ nets would discourage use (unless they have serious problems with user-acceptability). More robust nets would be unlikely to behave (in terms of use) like stored nets.

The effect of the availability of extant nets (stored or in use) on the rate at which new nets are taken into use and the effects of alternative stored or replacement nets on both the probability that a net is used on any one night and on the rate at which damaged nets are attrited, all need to be better understood. The fact that nets were not taken into use immediately in the Kenyan study population, suggests that households often store new nets away until they perceive a need for them (possibly to replace pre-existing nets when those become (too) damaged), but the extent to which this protects the nets from damage depends on different aspects of storage conditions, such as the presence of rodents (Koenker, unpublished). The existence of a stock of stored nets will likely dampen oscillations in coverage induced by intermittent mass distributions because there is a variable lag period between acquisition of a new net and putting it to use. As with continuous distribution, such dampening could have health benefits by smoothing the coverage spikes. It would imply that timing of net distributions is less critical than maintaining an adequate distribution rate of nets in the medium term. Simulations built on the NetCALC algorithm have suggested that pre-existing nets only need to be taken into account in planning distribution strategies when coverage is high [38], but this says nothing about the implication of (pre-existing) stocks of unused nets. Only if there is no replacement by newer nets do non-use and attrition increase vulnerability to malaria. The translation of the effects of a net on vectorial capacity (as quantified by Briet and colleagues [7]) into effects on infection rates and disease also depends on how many other people are using nets (and on the time of year) [39]. The present analysis supports the widespread belief that physical decay of nets is a major driver of attrition, but even the counterfactual analyses presented here cannot be used to make precise predictions of the effects of use or of stronger nets on attrition, because these are likely to depend on whether people have spare nets to fall back on.

## Conclusion

The model presented here extends the framework of earlier analyses of bed net population dynamics by considering physical integrity. This approach can contribute to understanding households’ net retention behaviors. It would need further extension to consider the dynamics of availability of other nets in the household and other factors (such as the level of nuisance biting by mosquitoes) that might modify the rate at which nets are taken into use. Focusing on durability contrasts with the strategy for improving LLINs by impregnating them with the synergist piperonyl butoxide (PBO) or a second, non-pyrethroid insecticide. In trials, such nets have reduced malaria burden more than pyrethroid only nets [40–42] (substantially in the western Tanzania study [41]). However, these nets are more expensive. It is possible that without a substantial increase in funding, the public health benefit from these new LLINs would be offset by a reduction in coverage and, therefore, use. There is a need for further data to better establish the physical durability of different net brands, which is a key determinant of cost-effectiveness.

## Supplementary Information

**Additional file 1.** Supplementary data description and analysis.

## Data Availability

The R code used for the main analyses and the corresponding data are available at: https://github.com/ThomasASmith/NetDurability. Other datasets used and/or analysed during the current study are available from the corresponding author upon reasonable request.

## Abbreviations

LLIN: Long-lasting insecticidal net
ODE: Ordinary differential equation
ITN: Insecticide treated net
WHO: World Health Organization
PMI: US President’s Malaria Initiative
pHI: Proportionate hole index
MCMC: Markov chain Monte Carlo
WHOPES: WHO Pesticide Evaluation Scheme
