# Supplementary material for: Incidence and consequences of damage to insecticide-treated mosquito nets in Kenya
Source: Malar J. 2021 Dec 20;20:476. doi: 10.1186/s12936-021-03978-7 (PMC8686568; doi:10.1186/s12936-021-03978-7)
Supplement: Supplementary file 1 — Additional file 1. Supplementary data description and analysis. [file 12936_2021_3978_MOESM1_ESM.pdf]

Additional File: Supplementary Data Description and Analysis.

Incidence and consequences of damage to insecticide-treated mosquito nets in Kenya

Thomas Smith[1,2]*, Adrian Denz[1,2], Maurice Ombok[3], Nabie Bayoh[3], Hannah Koenker[4], Nakul Chitnis[1,2], Olivier Briet[1,2], Joshua Yukich[5], John E. Gimnig[6]

*Corresponding author, Thomas-A.Smith@unibas.ch

[1] Swiss Tropical and Public Health Institute, 4051, Basel, Switzerland.

[2] University of Basel, 4001 Basel, Switzerland.

[3] Kenya Medical Research Institute (KEMRI), Kisumu, Kenya

[4] Tropical Health LLP, Baltimore MD, USA.

[5] Tulane University School of Public Health and Tropical Medicine, New Orleans, Louisiana, USA.

[6] Division of Parasitic Diseases and Malaria, Centers for Disease Control (CDC) and Prevention, Atlanta GA, USA

Supplementary data description

Table S 1 gives a breakdown of the recorded information on the causes of attrition and reasons why nets were absent at the time of survey.

Table S 1. Classification of nets by status at cross-sectional surveys

Use Status		Number of survey points
In use	In use last night	15,851
	Not in use last night	2,350
Not in use		5,542
Attrition	Destroyed-Burned by fire	728
	Discarded-Too torn[1]	706
	Discarded-Not killing mosquitoes	25
	Other	144
Absent	Lost/Stolen	1,169
	Given away or sold	807
	Household moved away	2,637
Total observations		29,959

Destructively sampled nets are listed under their status prior to being removed to the laboratory.
[1]The analysis of reported reasons for attrition considers only nets classified as 'Discarded-Too torn' as reported damage.

Posterior densities and correlations from MCMC fitting

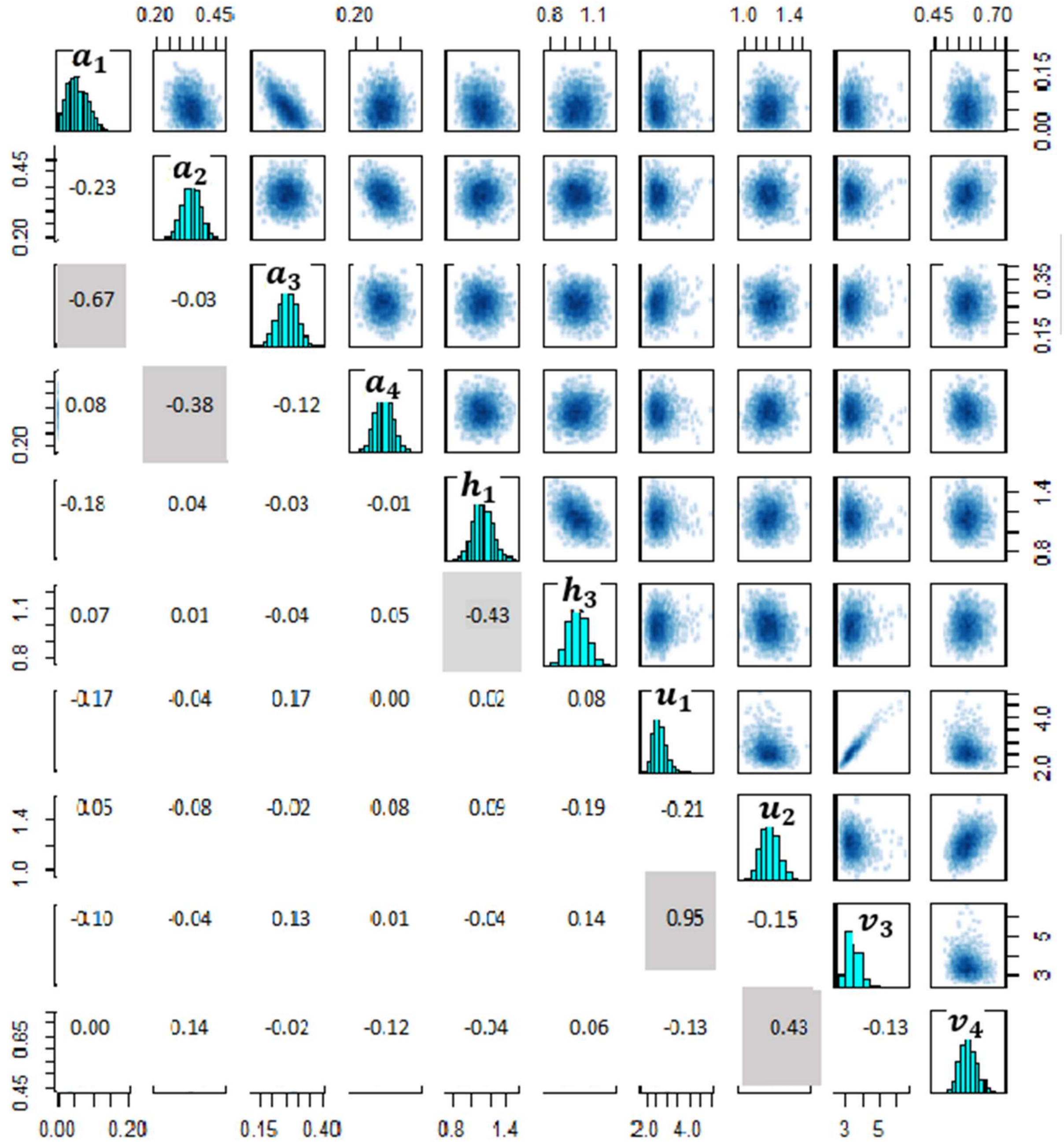

Figure S 1. Pairs plot of the Markov Chain Monte Carlo (MCMC) samples for ODE model parameters

The marginal distribution for each parameter is in the main diagonal. The entries above the main diagonal are scatters of paired samples from the posterior distributions. The entries below the main diagonal are the corresponding correlation coefficients. Correlation coefficients with magnitude greater than 0.3 are shown against a gray background.

Sensitivity analyses

Methods

The parameters of the ODE models were re-estimated with four distinct definitions of attrition (A1–A4), for different qualifying levels of damage (analysis A1_0), and for different assumptions about nets that were recorded as transitioning from damaged to undamaged (analysis A1_I) (Table S 2, Table S 3).

Table S 2. Sensitivity analyses

Analysis	Absent nets	Nets elsewhere	Damage cutoff	Coding of 'repaired' nets
A1 (Reference)	Attrition	Censoring	pHI=20	Damaged
A2	Censoring	Censoring	pHI=20	Damaged
A3	Censoring	Attrition	pHI=20	Damaged
A4	Attrition	Attrition	pHI=20	Damaged
A1R	Attrition	Recycled	pHI=20	Damaged
A1_0	Attrition	Censoring	pHI=0	Damaged
A1_I	Attrition	Censoring	pHI=20	Undamaged

A1 treats absent nets as attrition but nets elsewhere are not considered as attrition; rather they are treated as censored (i.e. having unknown status).

A2 considers as attrition only those nets that were reported as destroyed or repurposed (repurposed nets are included with *destroyed* nets for the purpose of this analysis). Intervals that ended with the net being sold, given away, or relocated were not included in this analysis (effectively treating such nets as remaining in the cohort. This model was used to estimate P_1, the proportion of destroyed nets that had been damaged before they were destroyed (see section ***Error! Reference source not found.***). This gave the value:

$$P_1 = 0.985.$$

A3 considers as attrition nets that were recorded as elsewhere, in addition to those recorded as destroyed, where elsewhere encompasses sale, giving away, or relocation. This model was used to estimate P_2, the proportion of all these nets that had been damaged before attrition according to this broader definition:

$$P_2 = 0.905.$$

The questionnaire responses reported in **Error! Reference source not found.** provide an estimate of P_3, the proportion elsewhere among absent and destroyed nets,

$$P_3 = 5687/(1750 + 5687) \; = \; 0.765.$$

P_4 is defined as the proportion of nets elsewhere that were damaged before they were taken away, and can be obtained from simple probability calculations, since:

$$P_2 = P_1(1 - P_3) + \; P_3 P_4,$$

which can be rearranged to give:

$$P_4 = \frac{P_2 - P_1(1 - P_3)}{P_3} = 0.880.$$

A4 considers as attrition all nets that were eligible to be followed up but were not present. In addition to destroyed and nets that were elsewhere, this includes nets that were *absent* for which there was no data, in those cases where the net did not reappear at a subsequent survey. This model was used to estimate P_5, the proportion of all nets that had been damaged before the survey where they did not appear. This gave the value:

$P_5 = 0.834.$

Table S 3. Classification of intervals analyzed by use status.

Initial status	Final status	Analysis			
		A1	A2	A3	A4
New	Last Night	2614			
Last Night	Last Night	9981			
Not Last Night	Last Night	736			
Not Use	Last Night	1705			
New	Not Last Night	349			
Last Night	Not Last Night	714			
Not Last Night	Not Last Night	259			
Not Use	Not Last Night	284			
New	Not Use	1106			
Last Night	Not Use	1745			
Not Last Night	Not Use	327			
Not Use	Not Use	1887			
New	Attrition	36	36	314	314
Last Night	Attrition	1267	293	1281	2255
Not Last Night	Attrition	151	44	196	303
Not Use	Attrition	485	177	796	1104

Recycling of nets

Treating absent nets as a mixture of destroyed and nets that were elsewhere, the mixing proportion was estimated by assuming the association between holes and the outcome (destruction or removal) to be the same in the absent nets as for those with explicit information about the outcome, and that this can be quantified by the odds ratio:

$$\Psi = \frac{P_4(1-P_1)}{P_1(1-P_4)} = 0.112.$$

The Markov property of the transition model justifies simulating nets that are elsewhere as returning either to compartment S_1 (if they are undamaged), or to compartment S_2 (if they are damaged). Recycling of absent nets was simulated using the A1 primary definition of attrition, but at each time, t, returning a proportion P_3 of attrition to the forward simulations, where the allocation between compartments S_1 and S_2 was determined from the proportion of attrition arising from damaged nets, $P_H(t)$, and the odds ratio, Ψ, where:

$$P_H(t) = \frac{a_2S_2(t) + a_4S_4(t)}{a_1S_1(t) + a_2S_2(t) + a_3S_3(t) + a_4S_4(t)}$$

The allocation between S_1 and S_2 was determined from the 2 x 2 table classifying the attrition at any given time (Table S 4). Defining x as the (time dependent) proportion of attrition that both have holes and will be recycled (to S_2):

Table S 4. Classification of attrition for simulating recycling

	Damaged	Not damaged
Recycled	x	$P_3 - x$
Destroyed	$P_H - x$	$1 - P_3 - P_H + x$

Where x is obtained as the solution of a quadratic equation, from:

$$\Psi = \frac{x(1 - P_3 - P_H + x)}{(P_3 - x)(P_H - x)}$$

So that the model equations (model A1R) become:

$$\frac{dA}{dt} = (a_1S_1 + a_2S_2 + a_3S_3 + a_4S_4)(1 - P_3)$$

$$\frac{dS_1}{dt} = v_3S_3 - (u_1 + h_1 + a_1)S_1 + \left(\frac{P_3 - x}{1 - P_3}\right)\frac{dA}{dt}$$

$$\frac{dS_2}{dt} = h_1S_1 + v_4S_4 - (u_2 + a_2)S_2 + \left(\frac{x}{1 - P_3}\right)\frac{dA}{dt}$$

$$\frac{dS_3}{dt} = u_1S_1 - (v_3 + h_3 + a_3)S_3$$

$$\frac{dS_4}{dt} = h_3S_3 + u_2S_2 - (v_4 + a_4)S_4$$

Where the parameter vector $\{h_1, h_3\ u_1, u_2\ v_3, v_4,\ a_1,\ a_2,\ a_3,\ a_4\}$ is that estimated with the A1 definition of attrition.

Results of sensitivity analyses

With the exception of the parameters measuring attrition rates, the fitted values for the parameter vector were similar for each of the analyses A1–A4, and for A1_0 and A1_I (Table S 5). This was reflected in the derived values given in ***Table S 6***, where substantial differences are seen only in the projected lifetimes of the nets. Simulation A1_0, in which the reference model (A1) was re-fitted, classifying nets with any holes as damaged (rather than requiring a PHI>20) gave very similar results to the original (A1). Similarly, the alternative coding of apparently repaired nets (A1_I), in which these were coded as undamaged at both start and end of the interval, made little difference to the projections from the model (Figure S 2).

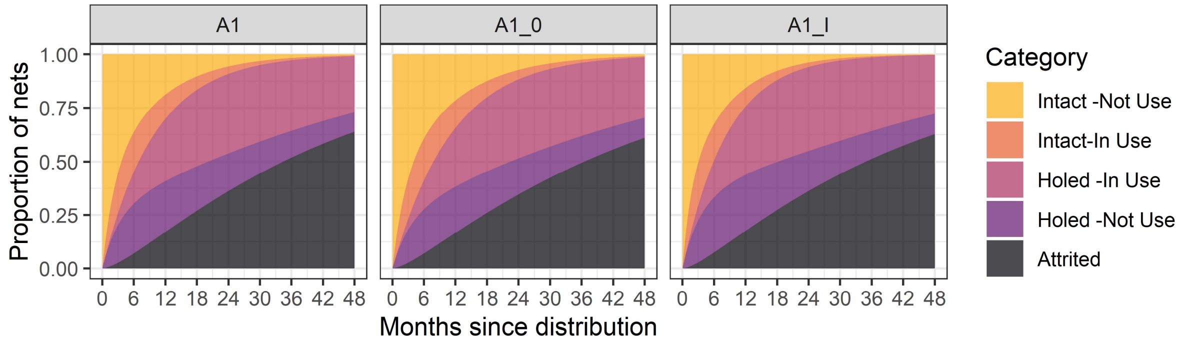

Figure S 2. Projections from models using alternative criteria for physical integrity.

The alternative definitions of attrition made considerable differences to the model predictions (Figure S 3). Analysis A2, which considers only explicitly destruction or repurposing as attrition, suggests a median net lifetime of 9.3 years (Table S 2); conversely, A3 and A4, which have more inclusive definitions of attrition, give rise to simulations with shorter lifetimes.

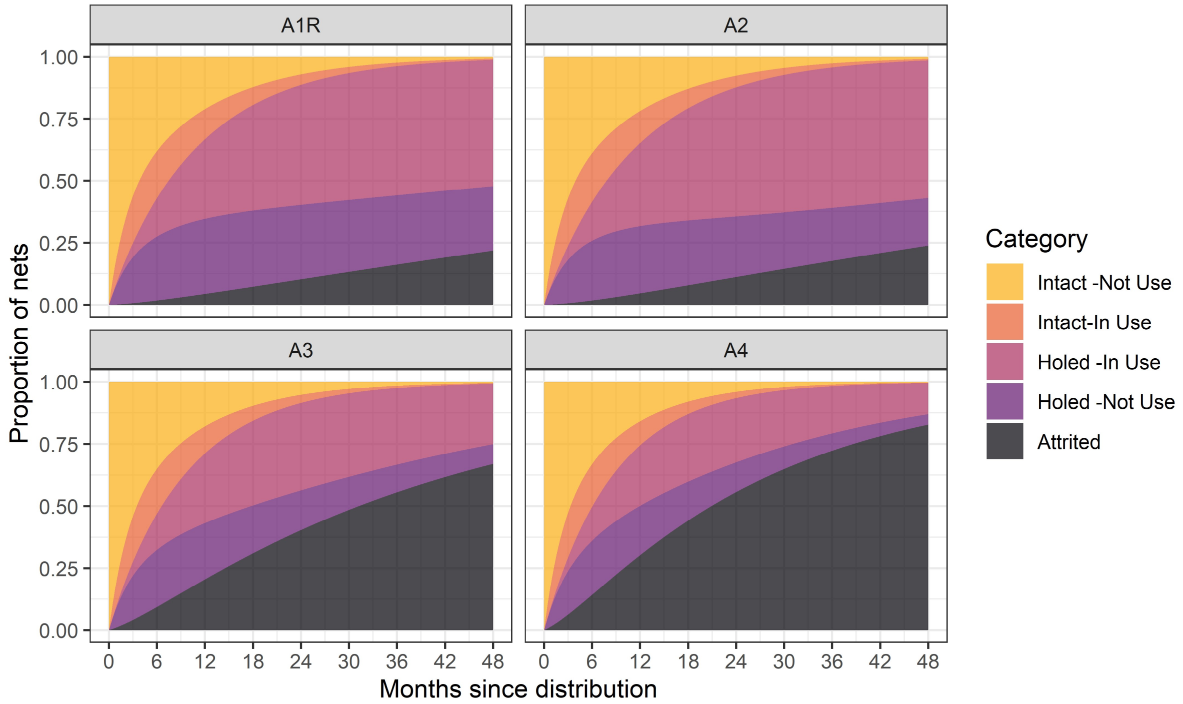

Figure S 3. Projections from models using alternative definitions of attrition.

The simulation with recycling (A1R) gives similar results to A2. The definition of attrition made little difference to the simulated proportion of nets in the undamaged states (Figure S 3), and in all simulations by the age of three years almost all nets are classified as damaged, so the differences between simulations mainly relate to the question of how often damaged nets that are absent, or which are stated to have been moved elsewhere, remain in use.

Table S 5. Parameter estimates for sensitivity analyses and net-type specific analyses.

Analysis	h_1	h_3	u_1	u_2	v_3	v_4	a_1	a_2	a_3	a_4	P_o
A1	1.18 (0.98, 1.42)	0.87 (0.76, 0.98)	1.67 (1.40, 2.01)	1.30 (1.13, 1.47)	2.77 (2.45, 3.22)	0.44 (0.39, 0.51)	0.04 (0.01, 0.09)	0.37 (0.29, 0.47)	0.26 (0.20, 0.33)	0.26 (0.23, 0.30)	0.60 (0.59, 0.61)
A1_O	1.01 (0.81, 1.26)	0.79 (0.67, 0.90)	1.59 (1.32, 1.90)	1.33 (1.17, 1.51)	2.55 (2.25, 2.96)	0.44 (0.39, 0.49)	0.04 (0.01, 0.10)	0.36 (0.28, 0.44)	0.33 (0.25, 0.39)	0.24 (0.21, 0.27)	0.65 (0.63, 0.66)
A1_I	1.39 (1.13, 1.68)	1.09 (0.95, 1.25)	2.30 (1.86, 3.06)	1.21 (1.06, 1.37)	4.14 (3.47, 5.33)	0.42 (0.37, 0.48)	0.03 (0.00, 0.08)	0.35 (0.27, 0.43)	0.33 (0.26, 0.41)	0.25 (0.22, 0.28)	0.60 (0.59, 0.62)
A2	1.11 (0.89, 1.38)	0.87 (0.75, 1.00)	1.63 (1.35, 1.97)	1.30 (1.13, 1.50)	2.83 (2.49, 3.32)	0.44 (0.38, 0.51)	0.01 (0.00, 0.03)	0.14 (0.09, 0.20)	0.02 (0.00, 0.05)	0.06 (0.04, 0.08)	0.60 (0.59, 0.62)
A3	1.24 (0.98, 1.52)	0.88 (0.75, 1.00)	1.64 (1.36, 2.00)	1.25 (1.08, 1.43)	2.86 (2.50, 3.36)	0.46 (0.39, 0.52)	0.12 (0.04, 0.19)	0.55 (0.44, 0.66)	0.12 (0.05, 0.19)	0.22 (0.18, 0.26)	0.60 (0.59, 0.62)
A4	1.25 (1.01, 1.51)	0.88 (0.77, 1.00)	1.67 (1.41, 2.01)	1.25 (1.10, 1.43)	2.80 (2.47, 3.23)	0.46 (0.40, 0.52)	0.17 (0.08, 0.26)	0.71 (0.60, 0.83)	0.36 (0.27, 0.45)	0.41 (0.37, 0.46)	0.60 (0.59, 0.61)
Dawaplus 2.0	0.62 (0.30, 1.07)	1.24 (0.93, 1.63)	0.91 (0.61, 1.28)	1.41 (0.93, 2.09)	1.70 (1.24, 2.28)	0.62 (0.44, 0.88)	0.03 (0.00, 0.11)	0.69 (0.41, 1.07)	0.42 (0.25, 0.61)	0.23 (0.12, 0.34)	0.65 (0.62, 0.68)
DuraNet	0.53 (0.15, 1.28)	0.56 (0.40, 0.77)	2.60 (1.55, 6.79)	0.46 (0.25, 0.79)	4.77 (3.17, 11.56)	0.30 (0.21, 0.42)	0.04 (0.00, 0.16)	0.22 (0.09, 0.45)	0.26 (0.11, 0.40)	0.22 (0.15, 0.31)	0.49 (0.46, 0.52)
Interceptor	0.61 (0.12, 1.20)	0.66 (0.44, 0.91)	0.58 (0.23, 1.07)	3.58 (2.37, 6.31)	1.91 (1.51, 2.40)	0.31 (0.18, 0.63)	0.07 (0.01, 0.17)	0.11 (0.01, 0.50)	0.21 (0.10, 0.33)	0.33 (0.25, 0.42)	0.59 (0.56, 0.63)
NetProtect	2.14 (1.26, 3.42)	0.85 (0.43, 1.20)	2.11 (1.08, 11.66)	1.63 (1.17, 2.27)	3.27 (2.24, 12.06)	0.76 (0.55, 1.05)	0.10 (0.01, 0.28)	0.36 (0.15, 0.60)	0.10 (0.01, 0.26)	0.26 (0.15, 0.38)	0.59 (0.55, 0.62)
Olyset	0.92 (0.53, 1.40)	1.41 (0.93, 1.95)	1.69 (1.17, 2.66)	0.77 (0.56, 1.04)	2.48 (1.62, 4.17)	0.80 (0.62, 1.04)	0.13 (0.02, 0.27)	0.35 (0.22, 0.52)	0.17 (0.02, 0.41)	0.27 (0.16, 0.38)	0.65 (0.62, 0.68)
PermaNet 2.0	0.45 (0.04, 1.06)	1.79 (1.26, 2.34)	2.08 (1.45, 5.01)	1.40 (0.81, 2.50)	1.83 (1.14, 5.73)	0.58 (0.36, 0.94)	0.12 (0.01, 0.29)	0.88 (0.50, 1.45)	0.22 (0.05, 0.44)	0.40 (0.23, 0.55)	0.61 (0.57, 0.64)
PermaNet 3.0	1.37 (0.86, 2.15)	0.71 (0.38, 1.02)	1.95 (1.08, 7.37)	2.06 (1.53, 2.73)	3.25 (2.24, 11.11)	0.29 (0.19, 0.43)	0.05 (0.01, 0.20)	0.15 (0.02, 0.38)	0.33 (0.15, 0.49)	0.19 (0.12, 0.26)	0.64 (0.61, 0.67)

Table S 6. Estimates of derived quantities for sensitivity analyses and net-type specific analyses.

	Median life of LLIN (years)	Reduction in net lifetime attributable to holes (years)	Proportion of lifetime of LLIN for which it is damaged	Proportion loss in net lifetime attributable to holes	Proportion of lifetime of LLIN for which it is in use[1]	Proportion of lifetime of LLIN for which it is in use[2]	Proportion of lack of use[1] attributable to holes
A1	2.86 (2.68, 3.08)	2.88 (1.77, 4.34)	0.78 (0.75, 0.81)	0.49 (0.38, 0.58)	0.60 (0.58, 0.62)	0.56 (0.54, 0.58)	0.13 (-0.08, 0.30)
A1_0	3.03 (2.82, 3.26)	1.83 (1.04, 2.82)	0.77 (0.74, 0.80)	0.37 (0.26, 0.47)	0.61 (0.59, 0.63)	0.57 (0.55, 0.59)	-0.09 (-0.28, 0.11)
A1_I	2.90 (2.69, 3.13)	2.35 (1.40, 3.61)	0.82 (0.80, 0.84)	0.43 (0.32, 0.53)	0.61 (0.59, 0.63)	0.57 (0.55, 0.59)	-0.03 (-0.25, 0.17)
A2	9.34 (7.91, 11.40)	8.59 *	0.91 (0.89, 0.92)	0.40 (0.29, 0.48)	0.69 (0.66, 0.71)	0.64 (0.62, 0.67)	-0.15 (-0.40, 0.03)
A3	2.60 (2.41, 2.82)	3.24 (1.77, 5.98)	0.78 (0.74, 0.81)	0.53 (0.40, 0.65)	0.60 (0.57, 0.62)	0.56 (0.53, 0.58)	0.20 (-0.04, 0.42)
A4	1.74 (1.64, 1.85)	1.23 (0.77, 1.96)	0.69 (0.66, 0.72)	0.44 (0.34, 0.55)	0.54 (0.51, 0.56)	0.50 (0.48, 0.52)	0.13 (-0.06, 0.30)
Dawaplus 2.0	2.64 (2.21, 3.12)	2.19 (0.72, 4.94)	0.67 (0.57, 0.76)	0.49 (0.38, 0.58)	0.52 (0.46, 0.59)	0.49 (0.43, 0.55)	0.13 (-0.06, 0.29)
DuraNet	3.90 (3.03, 4.99)	1.62 (-0.01, 4.44)	0.70 (0.53, 0.83)	0.46 (0.21, 0.63)	0.48 (0.39, 0.55)	0.44 (0.36, 0.51)	0.05 (-0.47, 0.40)
Interceptor	3.40 (2.80, 4.25)	3.32 (1.00, 9.42)	0.67 (0.47, 0.80)	0.29 (-0.02, 0.53)	0.63 (0.53, 0.70)	0.59 (0.49, 0.66)	0.01 (-0.52, 0.41)
NetProtect	2.73 (2.25, 3.30)	3.57 (0.63, 13.23)	0.85 (0.79, 0.90)	0.49 (0.27, 0.67)	0.59 (0.53, 0.64)	0.55 (0.50, 0.60)	-0.49 (-2.20, 0.05)
Olyset	2.73 (2.31, 3.26)	1.85 (0.38, 5.56)	0.77 (0.71, 0.83)	0.49 (0.38, 0.58)	0.44 (0.39, 0.50)	0.41 (0.36, 0.46)	0.31 (-0.45, 0.64)
PermaNet 2.0	2.01 (1.74, 2.31)	2.06 (0.69, 5.79)	0.64 (0.55, 0.72)	0.37 (0.26, 0.47)	0.60 (0.55, 0.66)	0.56 (0.51, 0.61)	0.34 (-0.08, 0.62)
PermaNet 3.0	4.00 (3.26, 5.12)	0.47 (-1.29, 2.71)	0.86 (0.80, 0.90)	0.43 (0.32, 0.53)	0.75 (0.70, 0.79)	0.70 (0.65, 0.74)	0.45 (0.14, 0.70)

[1]As defined by 'net in use'; [2]As defined by 'net in use last night';*interval estimates outside the simulated range.
